# Hydrothermal vents supporting persistent plumes and microbial chemoautotrophy at Gakkel Ridge (Arctic Ocean)

**DOI:** 10.3389/fmicb.2024.1473822

**Published:** 2024-10-03

**Authors:** Gunter Wegener, Massimiliano Molari, Autun Purser, Alexander Diehl, Elmar Albers, Maren Walter, Christian Mertens, Christopher R. German, Antje Boetius

**Affiliations:** ^1^MARUM, Center for Marine Environmental Sciences, University of Bremen, Bremen, Germany; ^2^Max Planck Institute for Marine Microbiology, Bremen, Germany; ^3^Alfred Wegener Institute, Helmholtz Centre for Polar and Marine Research, Bremerhaven, Germany; ^4^Department of Geosciences, University of Bremen, Bremen, Germany; ^5^Woods Hole Oceanographic Institution, Woods Hole, MA, United States; ^6^Institute of Environmental Physics, University of Bremen, Bremen, Germany

**Keywords:** hydrothermal vent, hydrogen oxidation, plume, sulfur oxidation, chemoautotrophy

## Abstract

Hydrothermal vents emit hot fluids enriched in energy sources for microbial life. Here, we compare the ecological and biogeochemical effects of hydrothermal venting of two recently discovered volcanic seamounts, Polaris and Aurora of the Gakkel Ridge, in the ice-covered Central Arctic Ocean. At both sites, persistent hydrothermal plumes increased up to 800 m into the deep Arctic Ocean. In the two non-buoyant plumes, rates of microbial carbon fixation were strongly elevated compared to background values of 0.5–1 μmol m^−3^ day^−1^ in the Arctic deep water, which suggests increased chemoautotrophy on vent-derived energy sources. In the Polaris plume, free sulfide and up to 360 nM hydrogen enabled microorganisms to fix up to 46 μmol inorganic carbon (IC) m^−3^ day^−1^. This energy pulse resulted in a strong increase in the relative abundance of SUP05 by 25% and Candidatus Sulfurimonas pluma by 7% of all bacteria. At Aurora, microorganisms fixed up to 35 μmol IC m^−3^ day^−1^. Here, metal sulfides limited the bioavailability of reduced sulfur species, and the putative hydrogen oxidizer *Ca.* S. pluma constituted 35% and SUP05 10% of all bacteria. In accordance with this data, transcriptomic analysis showed a high enrichment of hydrogenase-coding transcripts in Aurora and an enrichment of transcripts coding for sulfur oxidation in Polaris. There was neither evidence for methane consumption nor a substantial increase in the abundance of putative methanotrophs or their transcripts in either plume. Together, our results demonstrate the dominance of hydrogen and sulfide as energy sources in Arctic hydrothermal vent plumes.

## 1 Introduction

The chemistry of hydrothermal fluids differs strongly between and among mid-ocean ridges, back-arc spreading centers, and submarine volcanoes (Beaulieu and Szafranski, 2020; Diehl and Bach, 2020; Früh-Green et al., 2022). Hydrothermal fluids carry highly variable amounts of reduced components such as Fe^2+^, Mn^2+^, H_2_S, CH_4_, and H_2_ as well as complex organic molecules into the water column (Baker and German, 2004; Beaulieu et al., 2013). Upon cooling of hydrothermal fluids, the solubilities of different minerals change, resulting in the formation of metal sulfide and metal oxide deposits. At the seafloor, the chemical energy transported by vent fluids feeds diverse benthic microorganisms and their symbioses with invertebrates (Jannasch, 1985; Petersen et al., 2011). Accordingly, distinct assemblages of microbial taxa are associated with hydrothermal vents, including rock-associated, mat-forming, and invertebrate-hosted chemosynthetic microorganisms at the seafloor (Früh-Green et al., 2022). Some vent fields even host the largest biomasses known in marine environments (Dubilier et al., 2008; Jannasch, 1983; Van Dover et al., 2002). Because of the vigorous emission of hot fluids from some vents, the majority of reduced compounds escape the seafloor communities and form persistent plumes. These plumes may rise hundreds of meters above the seafloor and then spread laterally hundreds of kilometers away from their source (German and Seyfried, 2014; Schultz and Elderfield, 1997). While heat and fluid transports in hydrothermal plumes are relatively well understood, the influence of their variable chemical energy fluxes on the distribution of deep-sea life remains understudied, especially in polar regions (Bennett S. A. et al., 2013; Cathalot et al., 2021; Dick, 2019; Edmonds et al., 2003; McCollom, 2000). The total geothermal heat arising from Gakkel Ridge significantly warms the deep basin waters (Björk and Winsor, 2006), but little is known about the chemistry and microbiology of its hydrothermal vent fields (Edmonds et al., 2003). Thus, this study focuses on the influence of hydrothermal venting on microbial biodiversity associated with persistent plumes in the ice-covered Arctic Ocean.

Gakkel Ridge extends across the Arctic Ocean for a length of 1,800 km from Greenland to the Russian Shelf and has an ultraslow spreading rate of 8–13 mm per year (Cochran et al., 2003). An initial exploratory mission in 2001 discovered approximately 30 hydrothermal plumes along this ridge (Baker et al., 2004; Edmonds et al., 2003; Jokat and Schmidt-Aursch, 2007). This confirmed that hydrothermal venting along ultraslow spreading ridges is more abundant than previously anticipated (Bach et al., 2002; German et al., 1998; German et al., 1996). A combination of seafloor topography and rock sampling, coupled with plume detection, suggested that the majority of hydrothermal sources are associated with volcanic centers (Edmonds et al., 2003; Michael et al., 2003). Their occurrence under year-round ice cover has recently intrigued several international expeditions, including those conducted in conjunction with astrobiology and space science researchers, due to the potential analogy with hydrothermalism under ice on Earth to that possibly occurring on other celestial bodies, such as Saturn’s moon Enceladus (Hsu et al., 2015; Ramirez-Llodra et al., 2023; Seewald, 2017; Waite et al., 2017).

In their recent global analysis, Zhou et al., 2023 proposed that sulfur is the major energy substrate shaping the diversity of microorganisms in plumes (Zhou et al., 2023). In an earlier study of the first hydrothermal plume sampling at Gakkel Ridge, we discovered a novel Sulfurimonas type (*^U^Sulfurimonas pluma*) distinct from previously known microaerophilic types, whose abundance and global distribution in hydrothermal plumes seems to depend on the hydrogen availability (Molari et al., 2023). In contrast, a biogeochemical model suggests that the highly diluted chemical energy in hydrothermal plumes can support only minor chemolithoautotrophic activity (Cathalot et al., 2021). Here, we studied hydrothermal fields at the western and central Gakkel Ridge, and the relationship of energy sources, biogeochemical reactions, and microbial community compositions in their deep-water plumes. We analyzed the role of hydrogen, sulfur, and methane as energy sources available for the composition of the plume-hosted deep-water life, using a combination of *in situ* measurements, shipboard incubations, and sequencing of plume samples (16S rRNA genes and transcriptomes). Specifically, we tested to what extent hydrogen supports microbial life in the studied hydrothermal plumes.

## 2 Materials and methods

### 2.1 Bathymetry and maps

The Gakkel Ridge areas investigated here were partially previously mapped during the AMORE cruise in 2001, which used the former Hydrosweep DS-2 system of RV Polarstern (59 beams) and a Seabeam system of Healy (121 beams) (Thiede, 2002). During PS86 and PS101, we added survey tracks to fill up gaps in the AMORE bathymetry grid. RV Polarstern’s shipboard deep-sea multibeam echo sounder was an Atlas Hydrosweep DS-3. Its transducer frequency ranged from 13.6 to 16.4 kHz. The individual beam width was approximately 2.3°, which allowed a beam footprint of approximately 160 m at an area depth of 4,000 m. The swath width was set to 150% of the water depth throughout the entire mission. Peripheral sensors connected to the multibeam were a GPS Trimble receiver for positioning, an internal navigation system, and heave sensor system HYDRINS (Inertial Navigation System) for retrieving the ship’s roll, pitch, heading angles, and sound velocity keel probe. The acquired data were processed on board using Caris HIPS/SIPS Editor. The data were manually edited, filtered by applying matrix-based median filters, and exported to grids and xyz soundings. The resulting grids were produced at 100 m resolution. These grids were regularly updated as background layers for the real-time mapping tool GlobalMapper, which was used for the navigation and tracking of underwater instruments. All data are available in PANGAEA (links in the cruise reports), as well as further details on the methods reported below (Boetius, 2015; Boetius and Purser, 2017). The event labels are registered in the Earth System database www.pangaea.de and all further station information and data can be retrieved in open access from there.

### 2.2 OFOS photography transects

The Ocean Floor Observation System OFOS is an underwater camera system towed a few meters above the seafloor and equipped with both a high-resolution photo camera (iSiTEC, CANON EOS 5D Mark III) and a high-definition video camera (iSiTEC, Sony FCB-H11). The cameras are mounted on a steel frame (1,400 mm in length, 920 mm in width, and 1,350 mm in height), together with two strobe lights (iSiTEC UW-Blitz 250, TTL driven) four LED lights, and a USBL positioning system (Posidonia; iXblue) to track the position of the OFOS during deployments. Three laser pointers at a distance of 50 cm from each other were used to estimate the size of seafloor structures (Boetius, 2015). For PS101, the OFOS was upgraded with bathymetry technology to additionally mount side-scan and forward-looking sonar systems, referred to thereafter as the OFOBS system (Boetius and Purser, 2017; Purser et al., 2019).

### 2.3 Configuration and operation of the CTD and connected *in situ* pumps

The CTD rosette was operated with sensors recording conductivity as a measure of salinity, temperature, and density. The CTD was equipped with sensors for oxygen, sulfide, turbidity, and highly sensitive redox sensors (courtesy of Koichi Nakamura, National Institute of Advanced Industrial Science and Technology, Japan) and NOAA PMEL. Signals were processed and digitally transmitted to the ship. Additionally, Miniature Autonomous Plume Recorders (MAPR, supplied by the PMEL Earth-Ocean observation program of the National Oceanic and Atmospheric Administration; NOAA) were deployed along the ship’s wire and in particular at depths of the *in situ* pumps to enhance the resolution of measurements. Within the target areas, the CTDs were operated as towed yo-yo casts to detect and size plumes. In specific depths of interest (bottom water, below, in, and above the plume as identified by combined changes of temperature, turbidity, and E), the water column was sampled by closing one of the Niskin water samplers.

### 2.4 *In situ* filtration of seawater for molecular and geochemical analysis

To retrieve larger quantities of biomass and particles from plumes, background and reference water for molecular and trace metal analyses, we used large volume *in situ* pumps (WTS-LV04; McLane) equipped with polycarbonate filters (142 mm diameter; 0.2 μm pore size for molecular analyses) or hydrophilic polyethersulfone filters (HPWP1425; Millipore Express PLUS membrane filters, pore size 0.2 μm). In total, we analyzed the gene expression from 19 samples (Polaris) and 6 samples (Aurora). The filters were washed with trace metal-free solvents according to GEOTRACES protocols (Cutter et al., 2010). The pumps were programmed to operate at a maximum pump rate for 90 min. Pumps were installed on the CTD 10 to 60 meters above the CTD or at specific reference horizons. The pumps were placed in the plume for pumping time by placing the ship and lowering the CTD. Because filters have very small pore sizes, on average only 200 liters of water were filtered. For molecular analysis, pump heads were opened directly after retrieval, the filter was collected and sectioned into four equal pieces, which were shock-frozen in liquid nitrogen and stored at −80°C until analysis in the home laboratory. For trace metal analysis, pump heads were opened and complete filters were percolated with Milli-Q grade water (>18.2 MΩ) by using the *in situ* pumps in the manual operation mode. Filters were dried and stored at −20°C until analysis.

### 2.5 Determination of noble gas concentrations and isotopic ratios in water column samples

For the analysis of helium and neon isotopic composition, samples were collected in two ways from the Niskin bottles. All samples from the Polaris site and parts of the Aurora samples were collected by flushing a copper tube and closing the ends gas-tight with clamps. In addition, the Aurora site was sampled with an ampoule-based water sampler, where previously evacuated glass ampoules were filled half with the sample, and the headspace was directly available for analysis (Roether et al., 2013b). To compare the two approaches, 13 Niskin bottles were sampled with both methods. Because the background signals for ^3^He in this area are unknown, samples for CFC/Freon and Tritium analysis were taken around the plume signal to determine tritiugenic ^3^He as suggested by Roether et al. (2013a). These and the noble gas samples were analyzed and quality controlled in the Bremen Trace Gas Laboratory. After extraction, helium and neon were separated from other gases in cryo-traps at 25 and 14 K. Helium isotopes were analyzed with a high-resolution static mass spectrometer (MAP 215–50). The system is capable of resolving ^3^He from the mass-3 hydrogen species (HD and H_3_) leaking from metal walls. The high stability of the system provides an uncertainty of 0.5% for the ^3^He/^4^He ratio (Sültenfuß et al., 2009).

### 2.6 Determination of methane and hydrogen concentrations of water column samples

To determine methane and hydrogen concentrations of the CTD Niskin bottles, replicates of 40 mL of seawater were headspace-free sampled using a 60 mL syringe. Samples were heated to room temperature, and a 10 mL N_2_ headspace was applied. The syringes were vigorously shaken for 1 min to transfer the dissolved gases to the headspace. Methane was detected using gas chromatography coupled with flame ionization detection (Hewlett–Packard HP 5890 Series II Gas Chromatograph). Standard calibration was performed daily using NIST-traceable 100.0 ppm CH_4_ in N_2_. For hydrogen measurements, a Peak Performer gas chromatograph with a reduced compound photometer (Peak Performer 1, Peak Laboratories) with a 250 μL sampling loop was used and measured against 100 ppm H_2_ in air standards. At Polaris, we measured hydrogen and methane concentrations from 56 horizons. At Aurora, methane concentrations were determined from 30 samples.

### 2.7 Rates of dark CO_2_ fixation

Measurements of dark CO_2_ fixation (DCF) rates were carried out by applying a modification of the methodology previously described by Herndl et al. (2005) for deep-sea water. Water samples were collected from Niskin bottles using a sterilized silicon tube connected to sterilized 50-mL plastic syringes. Before the collection of the sample, the syringe and tube were washed with HCl (0.1 M), Milli-Q water, and three times with the Niskin seawater. Samples were stored in the dark at *in situ* temperature (−1 to −0.8°C) until the radiotracer injection, which occurred within 30 min after sampling. DCF rates were measured in 40-mL seawater in triplicate, with two formaldehyde-killed blanks supplemented with 40 μL of [^14^C]bicarbonate (1,380 kBq) and incubated in the dark at *in situ* temperature for 3 to 12 h for plume water and 48 h for background water. For assessing the concentration of added radiotracer, 5 μL of samples were added to 6 mL of scintillation cocktail (Filter Count, Perkin Elmer) and measured with a scintillation counter (TRI-CARB, Perkin Elmer). The incubations were terminated by the addition of formaldehyde (2% final concentration) to the samples. The samples were filtered onto 0.22-μm cellulose nitrate filters (Millipore) and rinsed three times with 10 mL of sterile-filtered seawater. Subsequently, the filters were exposed to fuming HCl (37%) for 2 h. After that, filters were transferred to a scintillation vial containing 6 mL of Filter Count, and the disintegration per minute (DPM) was measured using a scintillation counter. The DCF rates (μmol C m^−3^ d^−1^) are determined according to Reinthaler et al. (2010) (Equation 1):

(1) $$DCF=\left(DPMnet\times 1.05\times C_{DIC}\right)/\left(SA\times Tr\times V\times T\right)$$

with DPM*net* = DPM*sample* – DPM*blank* (*counts of the sample – counts of killed sample*), C_DIC_ is the dissolved IC in seawater (i.e., 2,100 μmol L^−1^), SA is the specific activity of [^14^C]bicarbonate (59 mCi mmol^−1^; Hartmann Analytic, Germany), *Tr* is the concentration of injected tracer measured at the start of incubation, volume V (cubic meter) and the time T (days). The factor 1.05 accounts for the discrimination of the ^14^C isotope in carbon fixation. A total of 86 samples (Polaris) and 75 samples (Aurora) were analyzed in these assays.

### 2.8 Time course experiments to determine methane and hydrogen consumption rates

Directly after recovery, we sampled water from dedicated Niskin bottles into multiple serum bottles (256 mL), which were sealed headspace-free with gas-tight rubber stoppers. In each experiment, 9 to 21 replicates were produced and immediately transferred back to *in situ* temperature (−1°C). Because oxygen concentrations were three orders of magnitude higher than that of potential electron donors, we based activity measurements on the consumption of the electron donors, not the oxygen. To measure potential methane and hydrogen consumption in reference and non-buoyant plume water samples, 250 μL of H_2_ and CH_4_ saturated seawater were added through the stopper to reach approx. 200 nM of both substrates. For other experiments, only hydrogen was added (see Supplementary Figure S3). At each sampling point, three of the replicate bottles were supplied with a headspace of 24.1 mL (1 mmol) synthetic air, in exchange for the same medium volume, which was removed with another syringe. After that, samples were warmed up to room temperature, and bottles were vigorously shaken for 30 s using a vortexer. To measure hydrogen from the headspace, 3 mL of sterile-filtered hydrogen/methane-free medium was injected into the bottle, and in exchange, 3 mL of headspace gas was pressed through connected tubing into the 250-μl sampling loop of the Peak Performer gas chromatograph (see above). For methane concentration measurements, 10 mL of gas phase was collected with a syringe, replacing 10 mL headspace with 10 mL of sterile medium. Methane concentrations were measured using a Hewlett–Packard GC and FID detection as described above. The resulting concentrations in ppmv were converted into nmol per liter seawater (nM). The hydrogen results are based on a total of 148 bottles of incubation (hydrogen), and all measurements were performed in technical replicates.

### 2.9 Determination of methane oxidation using ^14^CH_4_ tracer

Methane oxidation rate were determined in incubation experiments. Therefore we filled 156-ml cultivation vials completely with plume water and capped it with headspace-free with butyl rubber stoppers. We injected 20 µL of ^14^C-methane containing water through the rubber stopper (approx. 1 kBq per sample) and incubated the water at 0°C for 3 days. To stop potential microbial methane turnover, a headspace of 5 mL was supplied to all samples and 1 mL of 50% NaOH was added to the sample. In the home laboratory, concentrations of methane, methane tracer content, and produced ^14^C IC were determined following the protocol of Treude et al. (2005) Only a few of the incubated water samples showed ^14^C-IC values that were significantly elevated (>2 × standard deviation) compared to an average of ≥3 killed controls. A total of 46 samples (Polaris) and 107 samples (Aurora) were analyzed in these assays.

### 2.10 Determination of cell numbers

At sea, 500 mL of seawater was fixed with formaldehyde (2% final concentration) for 8 h at 4\u00B0C and filtered onto a 0.22-μm polycarbonate filter (47 mm diameter, Millipore). The filters were washed with sterile-filtered seawater and with 70% ethanol in MQ-water, and then after drying, they were stored at −20°C until further processing. In the home, laboratory cells were stained with DAPI (4′,6′-diamidino-2-phenylindole; Sigma-Aldrich). For a subset of samples, catalyzed reporter deposition fluorescence in situ hybridization (CARD-FISH) was performed using a specific probe for *^U^Sulfurimonas pluma* (Molari et al., 2023) and the SUP05 cluster (Meier et al., 2016). After that, cells were counter-stained with DAPI. All filters were enumerated under the epifluorescence microscope. For this, at least 1,000–2,000 DAPI-stained cells were counted and numbers were extrapolated for the filter size and seawater volume. A total of 12 samples (Polaris) and 34 samples (Aurora) were analyzed for total cell numbers. In total, 5 samples (Polaris) and 10 samples (Aurora) were analyzed with specific probes specific for *S. pluma* and SUP05, respectively.

### 2.11 Amplification and sequencing of 16S rRNA genes

Seawater samples for DNA analysis were filtered immediately after the retrieval of Niskin bottles, and the filtration was carried out in a temperature-controlled room (2°C) in the dark and did not exceed 1–1.5 h. At Aurora, 3 L of seawater was filtered onto 0.22-μm polycarbonate filters (47 mm diameter, Millipore) with a vacuum pump (N 022 AN.18; KNF, Freiburg, Germany). At Polaris, 8–10 L of seawater was filtered onto 0.22-μm Sterivex filters (Millipore) with a peristaltic pump (Masterflex; Cole Parmer, Vernon Hills, IL). After the filtration, the filters were stored at −80°C. DNA was extracted from the filter with DNeasy PowerWater Kit (MO BIO Laboratories, Inc., Carlsbad, CA, United States) following the protocol. Extracted DNA was stored at −20°C. Amplicon sequencing was performed at the Center for Biotechnology (CeBiTec laboratory, Bielefeld University). For the 16S rRNA gene amplicon library preparation, we used the bacterial primers 341F (5 ´-CCTACGGGNGGCWGCAG-3 ´) and 785R (5 ´-GACTACHVGGGTATCTAATCC-3 ´) (Herlemann et al., 2011), which amplify the 16S rDNA hypervariable region V3–V4 in Bacteria (400–425 bp fragment length). Sequences were obtained on the Illumina MiSeq platform (Illumina, San Diego, CA, United States) in a 2 × 300 bp paired-end run aiming for >50 000 reads per sample at CeBiTec Bielefeld, Germany, following the standard instructions of the 16S Metagenomic Sequencing Library Preparation protocol (Illumina, San Diego, CA, United States). The quality cleaning of the sequences was performed with the following software tools. The primer clipping was performed with the tool cutadapt (v1.9.1; Martin, 2011). Then the TRIMMOMATIC software v0.35 (Bolger et al., 2014) was used to remove low-quality sequences (SLIDINGWINDOW:4:20 MINLEN:300). The merging of forward and reverse reads was performed with the PEAR software (v0.9.6); setting a minimum overlap of 10 bp (Zhang et al., 2014). SWARM algorithm (v2.2.2; Mahé et al., 2014) was applied for clustering the sequences into operational taxonomic units (OTUs; local clustering threshold for operational taxonomic units set d = 1). The taxonomic classification of OTUs was based on the SILVA database, release 132 (Quast et al., 2013). The total number of sequences and OTUs generated in this study are reported in Supplementary Table S7. Sequences were deposited at the European Nucleotide Archive (ENA) under BioProject PRJEB48226 (accession numbers are reported in Supplementary Table S7). The sequences were archived using the brokerage service of the German Federation for Biological Data [GFBio; (Diepenbroek et al., 2014)]. A total of 21 (Polaris) and 16 (Aurora) samples were analyzed for these assays.

### 2.12 Metatranscriptomic analysis

After the recovery of *in situ* pumps, filters were immediately cut into six pieces, transferred to screw-cap tubes, frozen in liquid nitrogen for 5 min, and then transferred to −80°C. The RNA was extracted from three replicate filter sections using an RNase-free tube and the mirVana mRNA Isolation kit (Ambion GmbH, Germany). For DNA depletion, the extracted nucleic acids were treated with a TURBO DNA-free Kit (Ambion GmbH, Germany), and the RNA was purified and concentrated using RNeasy MinElute Kit (Qiagen GmbH, Germany). The RNA quantification, library preparation, and sequencing were carried out at CeBiTec laboratory (Bielefeld University) as described (Molari et al., 2023). The library was sequenced on a HiSeq1500 platform (Illumina, San Diego, CA), in 1 × 150 bases single-end runs, with a total number of reads per sample > 20 million. The adaptor sequence was removed and a read quality trimming with Q20 was performed using bbduk v34 from the BBMAP package and TRIMMOMATIC software v0.35 (Bolger et al., 2014). The trimmed reads were sorted in ribosomal RNA (rRNA) and non-ribosomal RNA (non-rRNA) using SortMeRNA software v2.0 (Kopylova et al., 2012) with the SILVA rRNA database. Statistics and ENA accession numbers are reported in Supplementary Table S8. The taxonomic classification of rRNA reads (1 million per sample) was assigned with phyloFlash software v3.0 beta 1 (Gruber-Vodicka et al., 2020) based on the SILVA database (release 132).

The non-rRNA reads of the transcriptome were *de novo* assembled using rnaSPAdes (Nurk et al., 2017) The genes were predicted on reconstructed transcripts using Prodigal [v.2.6.3 (Hyatt et al., 2010)], and the genes clustered using MMseq2 [v13.45111; (Steinegger and Söding, 2017) --min-seq-id 1 -c 0.80 --cov-mode 1 --cluster-mode 2]. Genes with length < 150 nucleotides were removed (*ca.* 12% of total genes). Taxonomic affiliation of the contigs was performed with Kaiju [v.1.9.2 (Menzel et al., 2016)] using a non-redundant protein sequences database (“nr”) and protein sequences from representative assemblies from NCBI BLAST (“refsed”). The functional annotation of genes was conducted using Pfam [v.35.0; (Mistry et al., 2021)] with hmmsearch HMMER [v.3.3.2; (Wheeler and Eddy, 2013)], NCycDB, a curated database for nitrogen cycling genes (Tu et al., 2019) with diamond v.2.0.15; (Buchfink et al., 2015), iron cycling genes FeGenie’s database (Garber et al., 2020) with diamond v.2.0.15, and Greening lab metabolic marker gene databases [Version 3; (Greening, 2021); with diamond v.2.0.15]. The metatranscriptomic reads were mapped to the gene sequences using bwa-mem2 v.2.2.1 (Vasimuddin et al., 2019) and converted in counts per gene with htseq-count script from HTSeq [v.2.0.2;(Putri et al., 2022)]. On average, 71 and 65% of reads from Polaris and Aurora metatranscriptomes, respectively, were retrieved by the catalog of genes (Supplementary Table S9). Gene read counts were divided by the length of each gene in kilobases (RPK) and reported as transcripts per million (TPM). We defined the genes highly transcribed in the plume, as those genes that have a level of transcription with centered log ratio value higher than 2 at PS101/188 (2,645 mbsl) and at PS86/055 (2,958 mbsl). A total of 19 samples (Polaris) and 6 samples (Aurora) were analyzed for these assays.

### 2.13 Data analysis

Non-parametric multidimensional scaling (NMDS) based on the Bray–Curtis dissimilarity index was used to depict differences in microbial communities based on counts of 16S rRNA genes, rRNA reads, and gene transcripts. All datasets were Hellinger transformed. The significance of differences between sites and seawater types was tested using the analysis of similarity (ANOSIM) (Clarke, 1993). The total effective number of species, exponential Shannon entropy, and inverse Simpson index were used to describe alpha-diversity (Chao et al., 2014) in each seawater type for Aurora and Polaris mounds. Beta-diversity between Aurora and Polaris plumes was quantified by calculating Jaccard dissimilarity based on rarefied (15,427 sequences) counts of 16S rRNA genes presence-absence (PA) transformed.

For the construction of the 16S rRNA phylogenetic tree, the sequences were aligned with MAFFT using the L-INS-i method with default settings (Katoh and Standley, 2014), and the alignment was cleaned with BMGE with default settings (Criscuolo and Gribaldo, 2010). Both programs were used on the Galaxy platform (Afgan et al., 2018). A maximum-likelihood-based tree was constructed using W-IQ-TREE (Trifinopoulos et al., 2016), first searching for the best substitution model (Kalyaanamoorthy et al., 2017), before evaluating branch support using 1,000 ultrafast bootstraps (UFBoot) and SH-aLRT branch test replicates. Phylogenetic trees for NiFe hydrogenase were constructed following the workflow described for the backbone 16S rRNA gene tree, with amino acids as coding sequence and MAFFT alignment method set to ‘auto’.

Significant differences in alpha-diversity, total cell counts, and CARD-FISH counts between seawater types and sites were tested by analysis of variance (one-way ANOVA) or by the non-parametric Kruskal–Wallis (KW) test when the ANOVA’s assumptions were not satisfied. Differentially abundant OTUs and 16S–18S rRNA reads (cDNA), and differential expressions of reconstructed transcripts were detected using the R package edgeR (Robinson and Smyth, 2007) at a significance threshold of 0.01 for Benjamini–Hochberg adjusted [BH; (Benjamini and Hochberg, 1995)] *p*-values (see Supplementary Tables S9, S10).

All statistical analyses and plots were conducted in R using the core distribution with the additional packages vegan (Oksanen et al., 2013), ggplot2 (Villanueva and Chen, 2019), and pheatmap (RRID:SCR_016418) (Kolde and Kolde, 2015).

## 3 Results

### 3.1 Identification of active vent fields at Gakkel Ridge

After the original detection of hydrothermalism across the ice-covered ultraslow spreading Gakkel Ridge in 2001 with temperature and turbidity sensors (Edmonds et al., 2003), this study had the objective to revisit plumes, assessing for the first time their sources at the seafloor and identifying potential chemoautotrophic life associated with the venting. The “Polaris” vent field was first visited in 2016 in the central part of the Eastern Volcanic Zone (Boetius and Purser, 2017) and “Aurora” in 2014 at the westernmost end of the Western Volcanic Zone (Baker et al., 2004; Boetius, 2015; Edmonds et al., 2003; German et al., 2022). In both areas, year-round full sea-ice coverage had prevented thorough studies of the source of the plumes prior to the RV Polarstern expeditions PS86 and PS101. Our main survey instruments to find the hydrothermal vents were ocean floor observing systems (PS86:OFOS; PS101:OFOBS) for seafloor morphology and geobiology (Purser et al., 2019) and a CTD (conductivity, temperature, depth) rosette that was equipped with highly sensitive redox and turbidity sensors, 24 Niskin bottle samplers, and *in situ* pumps. To survey the vent fields and sample their plumes, the icebreaker Polarstern had to drift with the ice floes across specific locations according to predicted trajectories (Boetius, 2015; Boetius and Purser, 2017).

**Figure 1:**
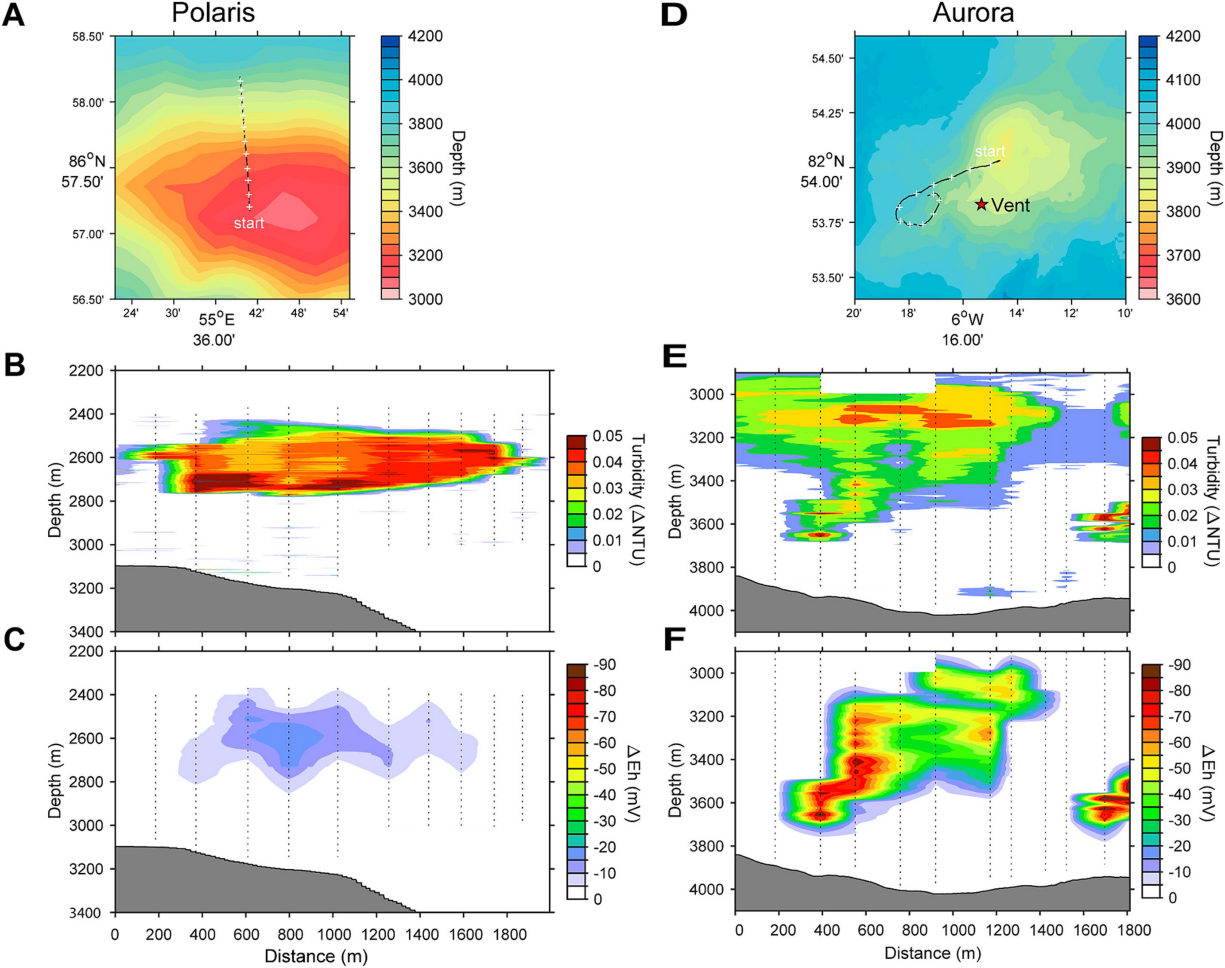
Locations and water column profiles of the studied vents. **(A,D)** Bathymetric maps and location of the Polaris and the Aurora vent. The star on the map indicates the position of vents observed by OFOBS. **(B,E)** Water column profiles for turbidity. **(C,F)** Water column profiles for plume-driven anomalies in redox potential (ΔEh).

During PS101, we visited the Eastern Volcanic Zone to investigate the source of a large hydrothermal plume hovering above the NW flank of a previously unnamed seamount discovered 15 years earlier (Baker et al., 2004; Edmonds et al., 2003). We named this structure “Polaris” (86°87.5’N, 55°42.4’E; 3,170 m water depth) (Figure 1A). In OFOBS surveys, we found indications for hydrothermal activity on the NW flank of the mount in the form of small vents with shimmering water. Some of the small vents contain orange precipitates or microbial mats. In the venting area, we detected a higher number of ophiuroids and polychaetes (Supplementary Figures S1A–E; for additional images, see Supplementary Table S1). The temperature sensor mounted on the OFOBS instrument recorded a temperature anomaly up to 1°C close to the seafloor, and high turbidity was visually detected above the seafloor by OFOBS, yet we did not find large chimneys or black smokers in the studied area. At Polaris, 13 CTD casts were performed in tow-yo mode to assess the size and extent of the plumes. The plumes and the surrounding water column were repeatedly sampled with Niskin bottles and high-volume *in situ* pumps.

During PS86, we discovered the Aurora field of black smoker vents at the southwestern flank of the mound (82°53.83’N; 6°15.32’W, in 3900 m water depth) (Figures 1D–F) during the PS86 expedition (Boetius, 2015). Subsequent expeditions (German et al., 2022; Ramirez-Llodra et al., 2023; Schlindwein, 2023) further studied the vent field with its giant, highly active chimneys. The chimneys were black to bright yellow-orange and released substantial amounts of particulate metal sulfides, visible as black smoke. The surroundings of the Aurora vent field showed basalt rocks, populated by glass sponges, amphipods, limpets, and snails of higher density than in the surroundings or at other mounds of this part of Gakkel Ridge (Supplementary Figures S1F–J, for additional images, see Supplementary Table S1; Boetius, 2015). Near the vent field, a number of cracks and fissures with diffuse vents and dead chimneys were discovered, the latter fully overgrown with sponges. The Aurora hydrothermal plume had a relatively strong turbidity signal that we could follow over a distance of approximately 2 km and for more than 800 m vertically (Figures 1D–F). Using OFOS, we were able to link the origin of this plume to the above-described field of black smokers (Supplementary Figure S1F; German et al., 2022). At Aurora, 14 CTD casts were performed, yet due to unpredictable ice flow movements, only approximately half of the casts reached the target area and allowed the sampling of the plume with Niskin bottles and *in situ* pumps.

### 3.2 Chemical energy in persistent non-buoyant plumes above vent fields

**Figure 2:**
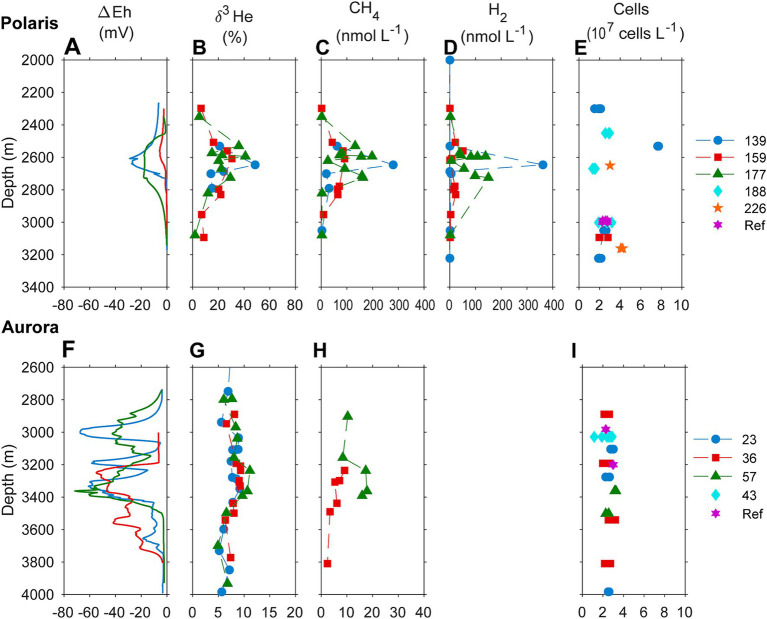
Biogeochemical signatures of the water column of **(A–D)** the Aurora and **(F–I)** the Polaris hydrothermal plume. **(A,F)** Redox potential measured as an offset of Eh value from background values; **(B,G)** helium isotopic composition measured as δ^3^H (%) against atmospheric helium as standard; **(C,H)** concentrations of methane in the water column; **(D)** hydrogen water column concentrations; and **(E,I)** cell numbers. Samples of different casts through the plume and reference sites are color-coded.

This study analyses the chemistry of large hydrothermal plumes associated with active venting from two volcanic seamounts. The plumes are rising several hundred meters above the rift valley of the Gakkel Ridge and were originally identified in 2001 (Edmonds et al., 2003). The non-buoyant component of the “Polaris” hydrothermal plume assessed by tow-yo CTD showed sharp anomalies in turbidity, redox values, and temperature extending from a depth of >2,800 meters below sea level (mbsl) to *ca.* 2,400 mbsl, i.e., ca 400–800 m above seafloor, with a horizontal stretch of over 1,200 m according to the turbidity signal (Figures 1B,C; Figure 2A). The CTD cast PS101-226 (86° 57.41’ N, 55 44.42′ E) intercepted the buoyant part of the plume closer to the seafloor (~2,900 m) with temperature anomalies up to 160 mK above ambient seawater (−0.8°C), suggesting further, more vigorous venting at the NW flank of the mound. Based on the plume rise height and the measured stratification of the water column, we calculate a vent power of 130 MW. At an endmember fluid temperature of maximal 270° C, which is typical for a low H_2_:CH_4_ ratio observed here (Table 1), this could translate into a water flux of at least 11,600 m^3^ per day (for calculation, see Supplementary material). The observed maximal redox anomaly was relatively low with −25 mV, and this redox signal disappeared faster than the turbidity signal (Figures 1B, 2A). The low redox anomaly likely results from the low concentrations of reduced iron and manganese in the plume below the detection limit (Table 1). The Polaris plume was rich in ^3^He, with δ^3^He values of up to 75% compared to atmospheric helium standards, indicating a substantial concentration of mantle-derived gas in the rising fluids (Figure 2B). The seawater samples from the buoyant part of the plume closer to the seafloor contained up to 300 nM CH_4_ and 360 nM H_2_ (Figures 2C,D). During the closure of Niskin bottles, temperature anomalies of up to 30 mK were measured. Hence, in all plume samples, the fluids were diluted by at least a factor of 1:9000 in the seawater (see Table 1). This suggests that the original vent fluids contained approximately 2.7 mM CH_4_ and 3.3 mM H_2_. Based on the calculated fluid flux rates, the Polaris vents would feed approximately 51,200 mol hydrogen and 43,200 mol methane per day into the plume. We detected neither dissolved nor precipitated iron (detection limit between 10 and 50 μM), and sulfide was analytically undetectable in the plume according to the Cline assay (detection limit ~10 μM). However, several plume samples had a noticeable sulfidic smell, which suggests sulfide concentrations greater than 100 nM. Therewith, sulfide total sulfide emissions from the Polaris vents could theoretically be in a similar range as hydrogen and methane emissions (Radford-Knoery et al., 2001).

**Table 1:** Physical–chemical parameters of hydrothermal venting at Polaris and Aurora.

	Polaris	Aurora
Max. values in the hydrothermal plume		
CH_4_ (nM)	300	30
H_2_ (nM)	360	300^c^
Max. ΔT (mK)	60	8
Dilution factor	9,000	46,250
Vent fluid endmember (calculated)		
Temp (°C)	≤270^a^	370^e^
Mn (mM)	b.d.	0.7
Fe (mM)	b.d.	6.8
CH_4_ (mM)	2.7^b^	1.4
H_2_ (mM)	3.3^b^	14^f^
Sulfide	>3 mM^c^	<10^e^
Total vent fluxes		
Power (MW)	130^d^	24^d^
Volume (m^3^ d^−1^)	11600^d^	1400^d^
Mn (10^3^ mol d^−1^)	-	0.85
Fe (10^3^ mol d^−1^)	-	8
CH_4_ (10^3^ mol d^−1^)	43.2	1
H_2_ (10^3^ mol d^−1^)	51.2	≥17

^aTypical temperature for observed CH4:H2 ratio; bcalculated based on dilution factors; cmin. Concentrations based on sulfidic smell and absence of free iron and the calculated dilution factor; dcalculated based on ocean physics and plume height, see methods; etypical black smoker temperature; f calculated from H2:CH4 ratios of typical black smokers.^

Based on the observed plume characteristics, we estimate that the vents have a power of at least 24 MW (for calculation see Supplementary material). Endmember fluids of volcanically hosted black smokers at ultraslow spreading ridges typically have temperatures of 370°C (Beaulieu and Szafranski, 2020; Diehl and Bach, 2020; Kumagai et al., 2008; Noowong et al., 2021), which would result in fluid fluxes of 1,400 m^3^ per day to feed the observed plume. In all retrieved samples, the temperature anomalies were low (< 8 mK), translating to dilution factors of ≥45 × 10^3^ for all samples. This high dilution of the Aurora plume is consistent with measured small ^3^He anomalies (δ^3^He values ≤10%) (Figure 2G). By contrast, the redox anomaly of the Aurora plume was much greater than that measured in the Polaris plume. The pronounced redox anomaly is consistent with high concentrations of dissolved iron and manganese in the plume, derived from the large black smoker field at Aurora (Figure 2F; German et al., 2022). *In situ* filtration of the water yielded a yellowish residue, suggesting partial precipitation of metal oxides and metal sulfides. The calculated concentrations of metals in the vent fluid endmember (6.8 mM Fe, 0.7 mM Mn) represent a conservative estimate, missing the fraction precipitated from the plume. Dissolved sulfide was not detectable, and the plume waters did not smell sulfidic. The plume contained up to 30 nM methane (German et al., 2022; Figure 2H), which translates to 1.4 mM methane in the fluid endmember. On board of PS86, we were not able to measure hydrogen concentrations. Typically, high-temperature vent fluids have a molar H_2_:CH_4_ ratio of approximately 10 or higher (Aquino et al., 2022; Charlou et al., 2002; Kumagai et al., 2008). Future studies should measure the endmember compositions. Based on the calculated fluid volume flux rates (see earlier), we estimate corresponding geochemical fluxes from the Aurora vents (Table 1) of approximately 17,000 mol H_2_, approximately 1,700 mol CH_4_ per day; >8,000 mol Fe per day (168 tons per year), and ≥ 850 mol of Mn per day (17 tons per year).

### 3.3 Hydrogen consumption in the plumes

**Figure 3:**
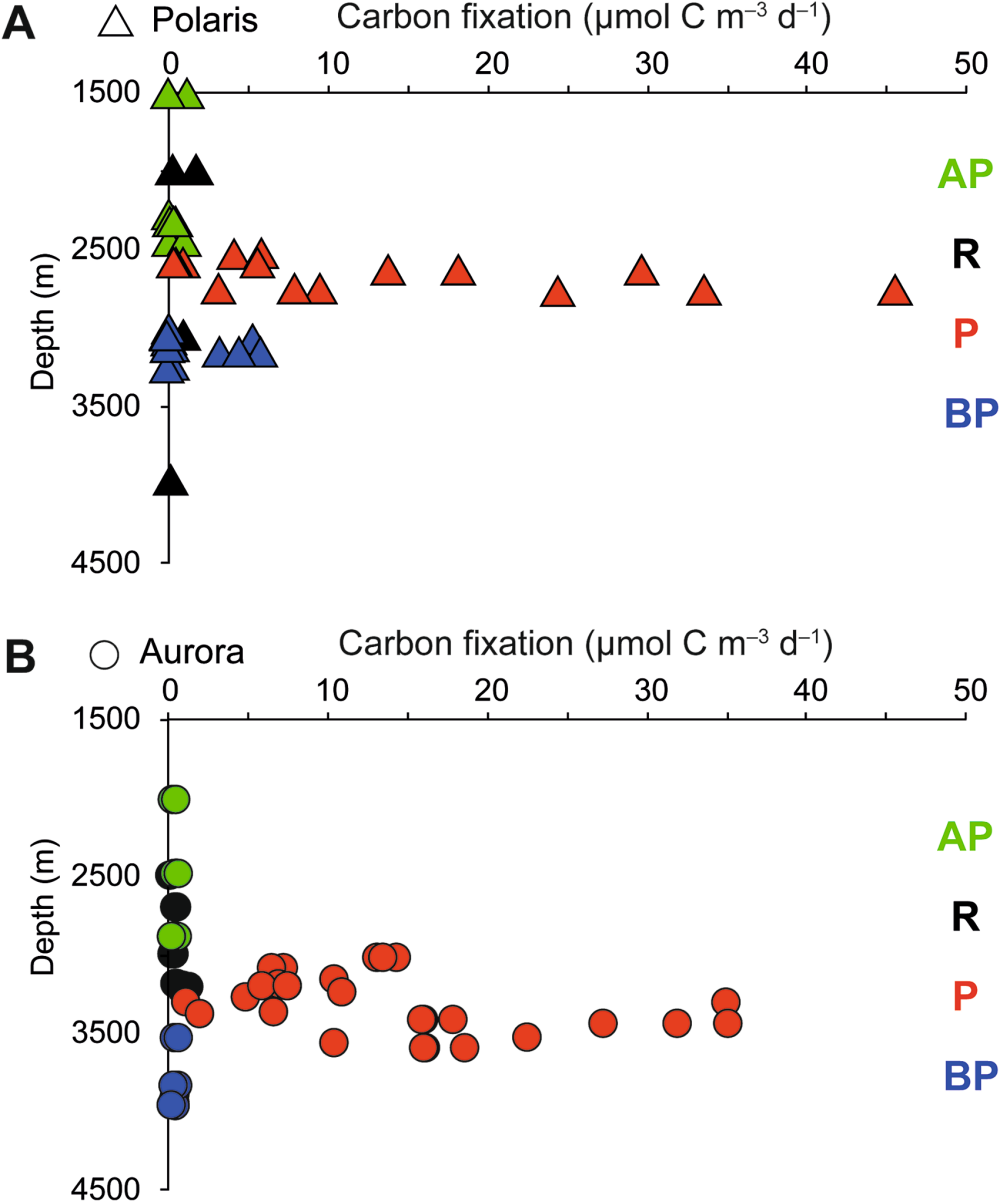
Stimulation of carbon fixation in **(A)** the Aurora and **(B)** Polaris plumes. Dark CO_2_ fixation (DCF) rates in deep-sea water collected above the plume (AP), in the plume (P), below the plume (BP), and at reference locations (R). Each data point represents a single rate measurement.

To quantify the influence of the energy sources on the carbon fixation by the non-buoyant plume microbiota, we collected water samples from the plume and local background waters (above and below each plume) and deep-sea reference water (>five nautical miles from the vents) from both, Polaris (*n* = 29) and the Aurora (*n* = 25) area. We incubated replicate samples of these waters with ^14^C-bicarbonate at *in situ* temperatures (−1°C) and measured the transfer of the radioisotope into the particulate carbon fraction (Reinthaler et al., 2010). Both reference waters distant from the vent, and local background water samples from above and below the plume, showed carbon fixation rates between 0.5 and 1 μmol C m^−3^ d^−1^ (Polaris *n* = 22 Aurora *n* = 15; Figure 3). These are typical values for dark carbon fixation in the oligotrophic deep ocean, as fueled by anaplerotic activity during heterotrophy and ammonium oxidation (Braun et al., 2021; Reinthaler et al., 2010). In contrast, plume water samples from both sites with notable *in situ* redox anomalies exhibited much higher carbon fixation rates between 5 and 45 μmol m^−3^ d^−1^ (Polaris *n* = 6; Aurora *n* = 10; Figure 3). At a mean carbon fixation of 13 μmol m^−3^ at Polaris, or 14 μmol m^−3^ at Aurora, and a carbon content of 10 fmol per cell (Fukuda et al., 1998), this would allow the growth of approx. 1.35 × 10^9^ autotrophic microorganisms per cubic meter of plume water each day. This translates into a maximal increase of cell mass of 60% per day compared to the background community. This value matches well with the moderately increased cell numbers (Kruskal–Wallis chi-squared = 0.18182, df = 1, *p*-value = 0.6698). Only one of the plume samples at Polaris (PS101/139) had three times elevated cell numbers compared to the average in the background (Figures 2E,I).

To evaluate the microbial metabolism associated with the non-buoyant plume waters, we compared hydrogen and methane concentrations in the water column and incubated replicate water samples from the hydrothermal plume and reference waters, (hydrogen data only available for Polaris). Within the different plume water samples, the concentrations of methane correlated with the helium isotope ratios, which suggests pure dilution and no consumption of methane during plume dispersion (Bennett B. et al., 2013). In contrast, the hydrogen concentrations dropped faster than the helium isotope ratios (Supplementary Figures S2A,B), indicating the microbial consumption of hydrogen. Consequently, the ratios of H_2_/CH_4_ dropped from values of 1.35 in the most concentrated plume samples close to the vent (characterized by higher δ^3^He values, CH_4_ concentrations, and/or temperature anomalies) to ratios near zero in more dilute samples (Supplementary Figures S2C–E). At Aurora, the methane: helium ratio is relatively constant, confirming that methane is not consumed (Supplementary Figure S2F).

**Figure 4:**
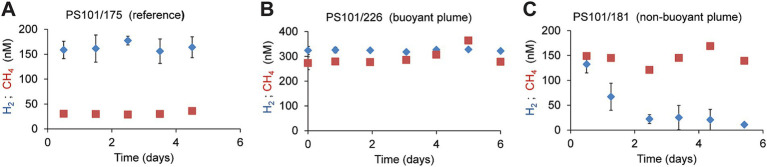
Development of hydrogen and methane concentrations in different water types. **(A)** In reference to waters incubated with hydrogen or methane **(B)** in buoyant plume water **(C)** in the outer, non-buoyant plume. In the reference and non-buoyant plume waters, hydrogen, and methane were added as sterile-filtered seawater equilibrated with methane/hydrogen-saturated water. Each symbol is a mean of *n* = 3 biological replicates, and error bars mark standard deviations.

To further compare the microbial consumption of methane and hydrogen, we incubated replicate bottles of background water (two different sites, 2,542 m; *n* = 24), buoyant plume water, (from one site; 3,010 m water depth; *n* = 19), and non-buoyant plume waters (7 sites, 2,722 m water depth; *n* = 90). We incubated water samples from the Polaris plume with low amounts of hydrogen and methane (for most samples, approx. 200 nM) at *in situ* temperatures of −0.8°C and *in situ* oxygen concentrations of 200 μM (Figure 4; Supplementary Figure S3), to mimic natural availabilities. In the background water (station PS101-175-5), concentrations of added hydrogen (165 nM ± 8) and added methane (31 nM; ±3) remained stable throughout incubation times of up to 5 days, i.e., neither of the energy substrates were consumed by microorganisms. (Figure 4A). Similarly, in the buoyant plume waters, the natural concentrations of hydrogen (323 nM) and methane (272 nM) remained stable (Figure 4B). In contrast, in samples of the non-buoyant plume, concentrations of added hydrogen decreased from 150 nM to 22 nM within only 2 days, whereas concentrations of added methane remained stable for the entire observation period of 5 days (Figure 4C). Four of six additional incubation experiments with non-buoyant plume water show clear hydrogen consumption (Supplementary Figure S3). Aerobic hydrogen oxidizers have growth efficiencies of up to 25% of the reducing equivalents released during hydrogen oxidation (Yu and Lu, 2019). An estimated average hydrogen oxidation rate of 30 μmol m^−3^ day^−1^ from all experiments would translate into the fixation of 7.5 μmol C m^−3^ day^−1^. This is approximately 50% of the carbon fixed in the plume. This amount might enable the growth of 0.75 × 10^9^ cells m^−3^ day^−1^.

### 3.4 Linking plume chemistry with microbial community activity

We investigated methane oxidation rates in the plume samples using highly sensitive ^14^C-methane radiotracer assays. Methane oxidation was not detectable, neither in the Aurora nor the Polaris plume nor in reference waters (lifetime of environmental methane ≥5 years; see Supplementary Table S2). To our knowledge, similar experimental quantifications of the fate of hydrogen and methane do not exist for any other non-buoyant vent plume to date. However, we were not able to test the influence of nanomolar amounts of reduced metals and sulfide on plume microbiota because of the lack of respective clean lab trace metal equipment and methods to measure low quantities of environmental sulfide at sea. In the Polaris plume, low metal concentrations result in the presence of free sulfide, allowing high microbial sulfur oxidation rates. In the Aurora plume, the excess of metals caused the formation of metal sulfides (black smoke), which will be less bioavailable.

**Figure 5:**
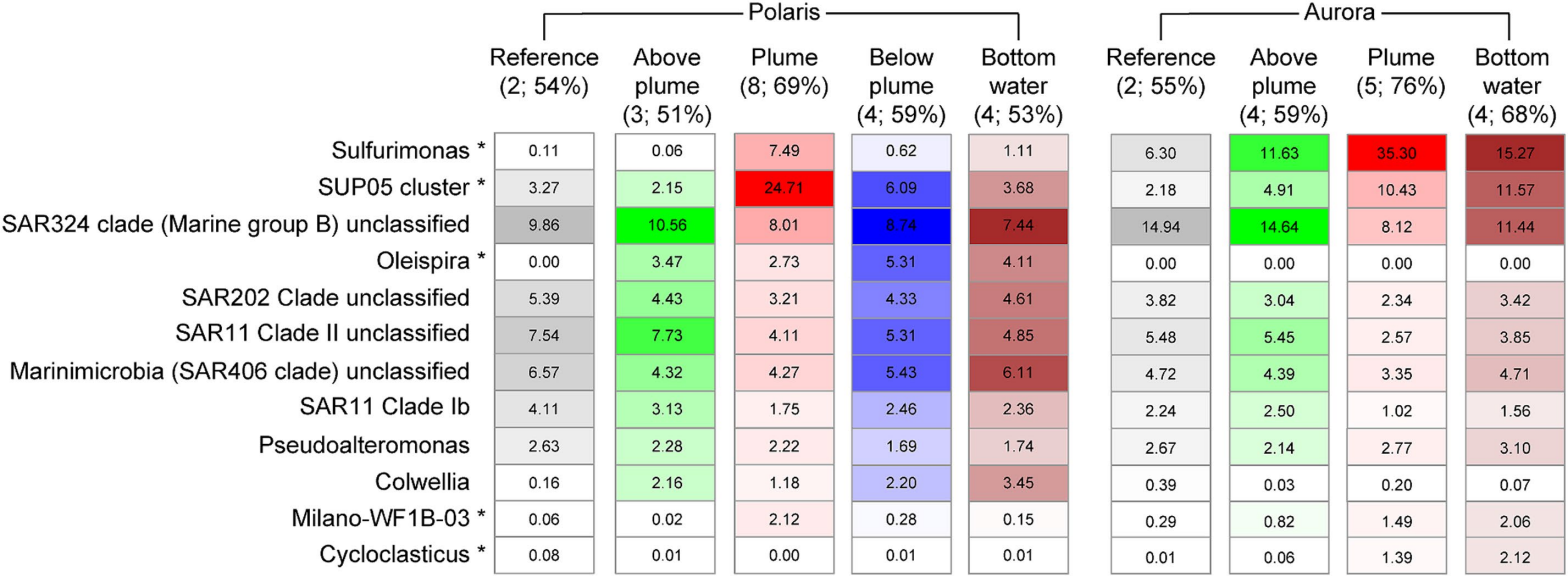
Relative abundance of microorganisms of different water masses at Polaris and Aurora based on 16S rRNA amplicon sequences. Relative abundance is calculated from amplified bacteria 16S rRNA genes. Only the 10 most abundant/significantly different abundant genera are shown (in brackets: numbers of samples and the sum of listed 10 taxa).

**Figure 6:**
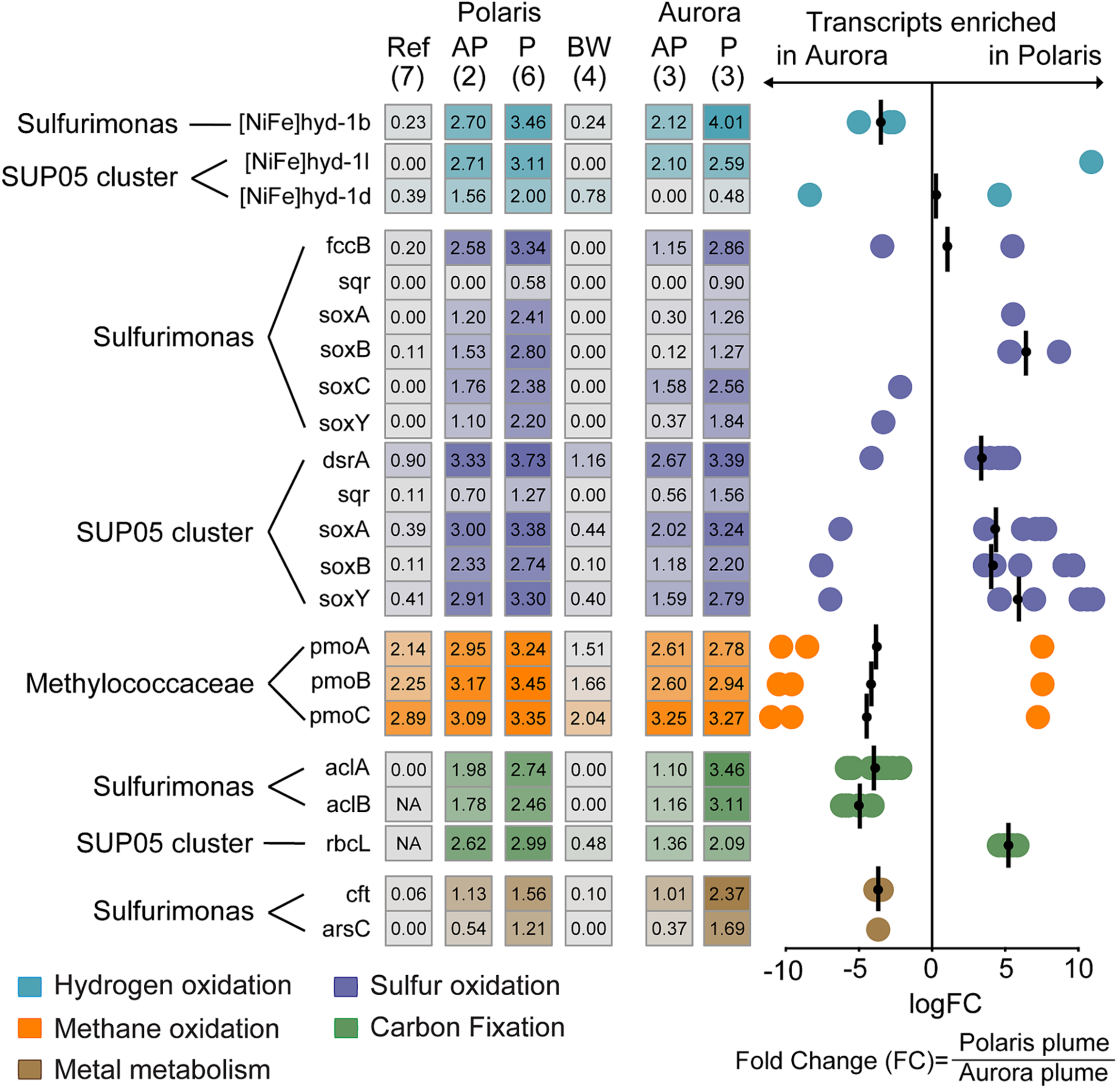
Expression of functional genes in different at Polaris and Aurora. Left: Heatmap depicts normalized transcription values at logarithmic scale for key functional genes of the hydrogen, sulfur, and methane metabolism assigned to Sulfurimonas, SUP05, and Methylococcaceae and their key genes for carbon fixation. In addition, the transcription values for metal transporters in Sulfurimonas in different water bodies are shown. In brackets: number of replications. Right: Enrichment of specific gene transcripts in plume samples of Aurora or Polaris, respectively. Each circle represents the fold change in for an individual gene encoding enzymes of a group specified with Pfam v.35.0 (Mistry et al., 2021). These genes do not necessarily belong to the same organism (see Supplementary Table S9 for details). The bars represent the geometric mean of the fold change in transcription for the genes encoding the same enzyme and were assigned to the same taxon. Negative values translate into enrichment in Aurora and positive values enrichment in Polaris. Genes: [NiFe]hyd1b/−l/1d: a large subunit of the different group 1 membrane-bound Ni,Fe hydrogenases; fccB, sulfide dehydrogenase flavoprotein chain; *sqr*, sulfide quinone reductase; *dsrA*, dissimilatory sulfite reductase alpha subunit; *aprA*, adenylylsulfate reductase alpha subunit; *SoxA*, sulfur oxidation protein subunit A; *pmoA*, particulate methane monooxygenase alpha subunit; *acl*A,B; ATP citrate lyase; alpha and beta *cbbL*: Rubisco large subunit. *Cft, arsC*: genes coding for metal metabolism.

To investigate the influence of the reduced compounds in the plumes on the abundance and activity of microorganisms, we analyzed the 16S rRNA genes and gene transcripts (here referred to as “active members”) extracted from plume, local background and reference waters (Polaris *n* = 19; Aurora *n* = 6; Figures 5, 6; Supplementary Figure S3; Supplementary Tables S7, S8). In the Polaris non-buoyant plume characterized by the highest carbon fixing activity, the most abundant taxon was SUP05 with 9% (PS101/139) to 47% (PS101/159) of total 16S rRNA gene sequences (average of 25% across all plume samples). In the background water and at reference stations, this taxon had a much lower relative abundance of 1–9% and 3–4%, respectively. At Polaris, Sulfurimonas contributed 4–19% in the non-buoyant plume compared to <1% in reference samples (Figure 5). Most of the other active fraction of microbial communities was similar in both plumes and in the background waters (Supplementary Figure S3). A difference between the non-buoyant plumes was the fraction of Sulfurimonas, which dominated the microbial communities at Aurora with 16 to 66% (PS86/055; Figure 5) of bacterial 16S rRNA gene sequences and 69 to 79% of all SSU rRNA reads (PS86/055; Figure 5; Supplementary Figure S4). SUP05 reached on average 10 and 8% in the Aurora plume, of total and active community members, respectively (Figure 5; Supplementary Figure S3).

The substantial enrichments of SUP05 and Sulfurimonas (i.e., *^U^S. pluma*) cells in Arctic hydrothermal plume waters were confirmed by microscopy applying specific oligonucleotide probes, reaching values of 6–12% and SUP05 2–20% Sulfurimonas cells, respectively, in the plume samples (Supplementary Table S3; Kruskal–Wallis chi-squared = 6.6, df = 1, *p*-value = 0.01). In background waters, Sulfurimonas still accounted for up to 2–10% of all SSU rRNA reads (PS86/074 at 2500 m), and SUP05 was 2%. These numbers—especially for *Sulfurimonas*—appear very high and suggest that Arctic deep-sea waters may be thoroughly mixed with hydrothermal waters from Gakkel Ridge (Edmonds et al., 2003), and that their mortality is very low. Furthermore, it remains to be investigated, if both taxa can maintain their energy needs by other electron donors.

To assess the impact of venting on the overall microbial community structure, we statistically analyzed the amplified 16S rRNA gene patterns. In both plumes, the increase of specific taxa resulted in a significant decrease of evenness as described by exponential Shannon entropy and inverse Simpson index, while the overall species richness of the water types was not affected (Supplementary Figures S5, S6; Supplementary Table S4). Aurora and Polaris plumes shared on average 36 ± 6% of bacterial 16S rRNA gene sequences with reference seawater, with lowest values at stations PS101/177 (25 ± 2%) and PS86/055 (29 ± 1%; Supplementary Table S5). The two plumes shared on average 32 ± 4% of the 16S rRNA genes, which is slightly lower than the value of 16S rRNA genes shared among the different non-plume waters (40 ± 1%; Supplementary Table S5). As previously reported by Molari et al. (2023), phylogenetic comparison of the Sulfurimonas SSU rRNA gene sequences from both hydrothermal plumes revealed two main distinct but closely related populations that share >99% SSU rRNA nucleotide sequence identity. Similarly, the analysis of the V3 − V4 region of the 16S rRNA gene of SUP05 revealed two related ribotypes (similarity >98%). One ribotype dominates at Polaris, and the other is more dominant at Aurora (Supplementary Table S6). These results were confirmed by the 98–100% identity between dominant OTUs of the 16S rRNA gene amplicons and 16S rRNA gene sequences from three metagenomic assembled genomes obtained from Aurora and Polaris water samples (Scilipoti and Molari, 2024). Based on phylogenetic analysis of their 16S rRNA, one of the two SUP05 strains clusters with SUP05 sequences from Pacific Ocean hydrothermal plumes, and the other species was more similar to SUP05 sequences from the Atlantic and the Pacific oxygen minimum zone (Supplementary Figure S7). In addition, some less abundant taxa show differential abundance. For instance, the Polaris plume was enriched in Oleispira, which include alkane oxidizers from the Arctic Ocean (Yakimov et al., 2003) and methane oxidizers of the Methylococcales cluster Milano-WD1B-03 (Supplementary Figure S4).

To analyze the metabolism of key community members, we analyzed the transcription activity for different functional genes. Transcripts for carbon fixation of Sulfurimonas via the reverse tricarboxylic acid cycle (i.e., *aclA* and *aclB*) are highly enriched at Aurora, whereas SUP05 transcripts for carbon fixation via the ribulose monophosphate pathway (*rbcL*) are more enriched at Polaris. This is in line with the dominance of either *Sulfurimonas* or SUP05 at Aurora and Polaris, respectively.

Genes for hydrogen and sulfur metabolism were highly expressed in the Polaris and the Aurora plumes, but the transcription levels differed between the two plumes and the abundant taxa (Figure 6). In both plumes, the hydrogenase [Ni,Fe]hyd1b assigned to Sulfurimonas was the highest expressed hydrogenase. This is the prototypical oxygen-sensitive hydrogenase of hydrogen oxidizers isolated from hydrothermal vents and other redoxcline environments (Adam and Perner, 2018, Lappan et al., 2023, Molari et al., 2023; Supplementary Figure S7), which supports the hypothesis of a dominant role of hydrogen oxidation at these Arctic vents. The dataset also contains transcripts of the groups 1d and 1 L [NiFe]-hydrogenase that belong to SUP05. Group 1d was highly enriched in the Polaris plume, but comparatively depleted in the Aurora plume. In contrast, the [NiFe]-hydrogenases group 1 L was similarly highly expressed in both plumes (Figure 6). These two hydrogenases most likely belong to two different SUP05 strains (Supplementary Figures S7, S8). Group 1d is the canonical enzyme for aerobic hydrogen oxidation, and it catalyzes hydrogen oxidation in the SUP05 symbionts of Bathymodiolus mussels from hydrothermal vents (Petersen et al., 2011). Hence, it is likely that the SUP05 of the Arctic plumes is also capable of growing on hydrogen. Recently, Lappan et al. (2023) reported that 1 L [NiFe]-hydrogenases are widespread in marine microorganisms; however, their function in many of these organisms remains unclear. SUP05 has been shown to grow on reduced sulfur compounds (Shah et al., 2017), and it was suggested that it also utilizes hydrogen (Anantharaman et al., 2013). Yet, not all SUP05 MAGs from hydrothermal plumes code for [NiFe]-hydrogenases (Dede et al., 2022; Zhou et al., 2023). Thus, it remains to be shown how widespread and important hydrogen metabolism is in pelagic SUP05 bacteria (Morris and Spietz, 2022).

At both sites, the transcripts of sulfur metabolism of SUP05 and Sulfurimonas are highly expressed. The highest enrichment of transcripts of the sulfur metabolism is found at Polaris. Here, the transcripts of SUP05 encoding the sulfur oxidation pathway, including sulfur oxidation to sulfate (*dsrA, aprA*) and thiosulfate oxidation to sulfate (*soxA, soxB*), belonged to the most enriched functional gees (Figure 6). In addition, transcripts of sulfur metabolism assigned to Sulfurimonas are substantially expressed, which points toward the potential of *S. pluma* to metabolize and grow on sulfur compounds. However, the dominance of Sulfurimonas in the hydrogen-rich Aurora plume and the dominance of SUP05 in the sulfur-rich Polaris plume combined with the transcriptomic data suggest different substrate affinities of both organisms and therefore potential niche separation as reported for other marine environments (Meier et al., 2017; Rogge et al., 2017).

Puzzled by the lack of evidence for microbial oxidation of methane, we also looked for the transcription of methane monooxygenases (*pmo*). Surprisingly, the pmo expression was relatively high in all water samples, and only in the Polaris plume some subunits were enriched compared to measurements in other water bodies (Figure 6). The relative abundance and activity of potential methanotrophs were low (≤ 2%; Figure 5; Supplementary Figure S4). These findings suggest that methanotrophs do not profit from the elevated methane concentration in the non-buoyant plumes, which has been predicted previously (Reed et al., 2015). This fits to the apparent lack of methane consumption in the plume as determined by the gas ratios and the incubations with ^14^C methane radiotracer. Moreover, we did not identify enrichment of transcripts related to iron oxidation within the pool of highly transcribed genes at Polaris and Aurora, suggesting minor importance of microbial metal oxidation (Dick, 2019; Zhou et al., 2023). Only some metal-related metabolic genes assigned to *Sulfurimonas* (*cft, arsC*) were highly transcribed in the plumes, matching the physiology of *Sulfurimonas* in their need for particulate metals (Molari et al., 2023).

## 4 Discussion

Our geobiological surveys of seamounts of the Western and Eastern Volcanic Zones resulted in the identification of persistent hydrothermal activity linked to large non-buoyant plumes in the deep Arctic Ocean. At both sites, i.e., Aurora and Polaris, we detected the plumes at the same sites as described in 2001 (Edmonds et al., 2003), showing significant turbidity and temperature anomalies compared to the background. At the Aurora mound, we tracked down the plume to a typical high-temperature black smoker field, where metals co-precipitate with sulfide. We calculated emissions of 168 tons of iron per year and similar amounts of sulfide from the Aurora field and observed the formation of large chimneys and iron–sulfur precipitates at the seafloor (Supplementary Figures S1F,H). At Polaris, we located the source of the plume at the previously described location at the NW axial volcanic ridge and detected a mound with substantial hydrothermal features. Yet the many small clear-water vent chimneys observed (20–30 cm in height) and the fissures between the rocks are unlikely the source for the large plume rising up to 800 m into the water column (Figure 1). The lack of iron in the overlying plume suggests a low-temperature type venting. According to preliminary seafloor observations, the hydrothermal areas hosted a higher number of different invertebrates than the surrounding area (e.g., accumulations of filter feeders, polychaetes, mat-type precipitates, and crustaceans; Supplementary Figure S2), yet it remains to be assessed if any of the different macrofauna species benefit directly or indirectly from microbial chemosynthesis.

Due to the low stratification of the Arctic Ocean’s deep waters, the formed hydrothermal plumes increased up to 900 meters in the water column. In the deep buoyant part of the plume, the numbers of potentially autotrophic microorganisms were low, and at Polaris, the water did not show immediate hydrogen oxidation activity. We explain the absence of microbial activity in the buoyant plume with the short rising period of approximately 1 h (Reed et al., 2015). Within this period, the ambient microbial communities are unlikely yet to respond to the presence of hydrogen or other reduced compounds. Moreover, most microorganisms transported from the vent chimney are likely thermo- or mesophilic and not adapted to the plume temperatures of −0.8°C. Thus, the high carbon fixation rates of up to 45 μmol m^− 3^ in the non-buoyant hydrothermal plumes are best explained by a specific functional microbial assemblage able to adapt to this extreme niche in the central Arctic Ocean. All samples showing an increased carbon fixation also showed a specific microbial community composition, selecting for chemosynthetic bacteria. The very sluggish tidal current regime of the deep Arctic Ocean can retain the same water mass for relatively long times above the vents.

At Polaris, the most abundant and active taxon is SUP05. SUP05 is widely distributed across different pelagic environments (Canfield et al., 2010; Glaubitz et al., 2013). Recent analyses based on amplified 16S rRNA genes and metagenomic analyses revealed that non-buoyant plumes are generally enriched in globally distributed SUP05 clades, which are recruited from background waters (Anantharaman et al., 2013; Dede et al., 2022; Dick, 2019; Dick et al., 2013; Lesniewski et al., 2012). Based on their dominance in most studied plumes to date, it was concluded that their specific deep-water niches are low-temperature, high-oxygen waters with low concentrations of reduced sulfur species (Dede et al., 2022; Meier et al., 2017). Hence, the microorganisms were exposed to physiologically relevant concentrations of vent-derived energy sources. Polaris and Aurora represent the coldest habitats of SUP05 so far found in the ocean. Still, their 16S rRNA genes cluster with sequences from strains in the Pacific and South Atlantic deep waters. At Aurora, *Ca. S. pluma* was more dominant. Here, hydrogen was the by far most abundant energy source, suggesting that the availability of hydrogen is a factor selecting for this group. Due to the precipitation of iron sulfide, reduced sulfur phases were only available in minor quantities; hence, the abundance of SUP05 increased only moderately. The use of hydrogen as the main energy source was supported by the highest expression of the hydrogenase in *Ca. S. pluma*, and the presence of *Ca. S. pluma* in other hydrogen- or metal-rich hydrothermal plumes (Molari et al., 2023). All previous cultures of Sulfurimonas showed a preference for suboxic conditions (Han and Perner, 2015). So far, it is not fully resolved how *Ca. S. pluma* thrives in oxygen-saturated Arctic seawater, but modifications in the reductive tricarboxylic acid pathway seem to have a role in this adaption (Molari et al., 2023).

The biogeochemistry of the hydrothermal vent plumes is also largely reflected in the gene expression patterns extracted from the plume waters. In the hydrogen-rich and sulfur-depleted Aurora plume, the hydrogenase of Sulfurimonas is the most expressed gene, suggesting the dominance of hydrogen oxidation by *S. pluma*. However, based on substantially expressed pathways for elemental sulfur oxidation (Sox pathway) and potentially sulfide oxidation (non-canonical flavocytochrome c), *Ca. S. pluma* may also be capable of growing on sulfur compounds. To test this hypothesis, further experiments and cultures are required.

In contrast, at Polaris, the genes of the oxidative sulfur metabolism *dsr, apr,* and the *sox* complex were enriched and linked to the higher bioavailability of sulfur. Interestingly, the sulfide quinone reductase (*sqr*) that encodes the enzyme for the activation of hydrogen sulfide was not strongly enriched. This may indicate that much of the hydrogen sulfide is oxidized chemically, and the Arctic plume microbiota rather thrives on the products, i.e., elemental sulfur and thiosulfate. The highly expressed *dsr*, *apr,* and *sox* genes belong primarily to SUP05, supporting the hypothesis that SUP05 relies on sulfur species as its main substrate (Anderson et al., 2013; Rogge et al., 2017). Because SUP05 also expresses hydrogenases, it might also utilize hydrogen in competition with *Ca. S. pluma*.

Altogether, hydrogen emissions appear responsible for a dominant fraction of microbial biomass production at the Polaris and the Aurora vents, respectively. Sulfur oxidation provides another important energy source in both vent plumes, similar to what is known from temperate vent environments (Zhou et al., 2023). Yet, our results showed that in the Arctic hydrothermal vent plumes, the sulfur-oxidizing community is less diverse than in other hydrothermal plumes (Zhou et al., 2023). Most interestingly, the bacteria selected by the persistent plume environment are represented in the background deep waters, even far away from the vent sites. It remains to be studied how they can survive in the absence of their main electron donors. Our study further confirms that sulfur speciation has a great influence on the bioavailability of sulfur. A co-occurrence with high metal loads in the endmember limits the bioavailability of this energy source.

Methane has been often suggested to play a role as an energy source to plume microorganisms, based on the widespread presence of aerobic methanotrophs, e.g., in 16S rRNA gene surveys as well as based on functional genes (Anantharaman et al., 2016; Lesniewski et al., 2012). However, in the plume waters studied here, the relative numbers of methane oxidizers such as Methylomonaceae were not significantly increased compared to surrounding waters (Figure 5). Despite notable transcription of the *pmo* genes both in the plume and in the background waters, we could not detect methane oxidation (Figure 4; Supplementary Figures S2, S3; Supplementary Table S2). Cultured methanotrophs show relatively high half-saturation constants for methane in the range of 2 to 12 μM (Bender and Conrad, 1992; Knief and Dunfield, 2005). In the plumes, methane rapidly diluted to low nanomolar concentrations, and together with temperatures below 0°C, this may be the reason, why methane-consuming microorganisms do not grow, even though methane oxidation is thermodynamically favorable. We conclude that methanotrophs may not be able to grow fast enough in these icy oceans to exploit the methane enrichments observed. Other vent plumes in warmer waters, such as those of the Juan de Fuca Ridge, show comparatively higher methane consumption (Cowen et al., 2002; de Angelis et al., 1993).

The location and exploration of vents and retrieving endmember fluids of vents in Arctic oceans remains technologically challenging. However, this study shows that relatively few chemical analyses, experiments, and simple community descriptions can already provide insights into the nature of the venting present. Conversely, it is also possible to predict plume community compositions from the fluid endmember chemistry. The vigorous venting found at Aurora and Polaris releases large amounts of geothermal energy, provides a source of rare elements into the deep ocean, and fuels a specific community, apparently hydrogen and sulfur-based microbial growth. Further studies are needed to resolve the extent of venting and its influence on the deep-sea microbiome and fauna across the Gakkel Ridge.

## Data Availability

The datasets presented in this study can be found in online repositories. The data presented in this study have been deposited at the European Nucleotide Archive (ENA) under the project number PRJEB48226. The 16S rRNA gene amplicon data can be found under the accession numbers ERR7132482-ERR7132512. The metatranscriptomic data was deposited under the accession numbers ERR7132513-ERR7132538. Further information can be found in the article/Supplementary material.
