# Supplementary material for: Hydrothermal vents supporting persistent plumes and microbial chemoautotrophy at Gakkel Ridge (Arctic Ocean)
Source: Front Microbiol. 2024 Oct 3;15:1473822. doi: 10.3389/fmicb.2024.1473822 (PMC11484012; doi:10.3389/fmicb.2024.1473822)

Supplementary Material

1 Supplementary text

Calculation of energy and mass fluxes from the Polaris and Aurora vent

Hot, buoyant fluid from hydrothermal vent ascents as a plume while entraining ambient fluid. In a stratified ocean, the plume rises until a height is reached where the diluted fluid has the same density as the surrounding water. The rise height z_{max} depends on the buoyancy flux F from the source and the stratification N^2. It can be expressed as

(1) $z_{max} = 5.0\ (F / \pi)^{1/4}\ N^{-3/4}$ (111).

Using the vent source volume flux Q_v, the buoyancy flux F is defined as

(2) $F = g\ Q_v\ [\alpha\ (\theta_v - \theta_b) - \beta\ (S_v - S_b)]$,

where g is the gravitational acceleration, α is the thermal expansion coefficient, and β the haline contraction coefficient. Assuming that the emanating fluid has the same salinity as the environment and neglecting the background temperature θ_b that is close to zero degrees Celsius, Eq. (2) can be used to estimate the vent's output

(3) $Q_v = F / (g\ \alpha\ \theta_v) = P / (c_p\ \rho_0\ \theta_v))$.

and

(4) $P = c_p\ \rho_0\ \alpha^{-1}\ g^{-1}\ F\ = c_p\ \rho_0\ \alpha^{-1}\ g^{-1}\ \pi\ (z_{max} / 5)^4\ N^3$

using

$c_p = 3890\ J\ kg^{-1}\ K^{-1}$
$\rho_0 = 1000\ kg\ m^{-3}$
$\alpha = 1.3 \times 10^{-4}\ K^{-1}$
$g = 9.8\ m\ s^{-2}$

gives

	Polaris	Aurora
Rise height z_{max} (m)	600	800

Stratification N^2 (s^{-2})	1.63×10^{-7}	0.25×10^{-7}
Vent power P (MW)	130	24
Vent temperature θ_v (°C)	250	370
Volume flux Q_v (L s^{-1})	135	17

McDougall, T. J. (1990). Bulk properties of “hot smoker” plumes. Earth and Planetary Science Letters, 99(1-2), 185-194.

2 Supplementary Figures

Supplementary Figure 1. Photographs of the Polaris and the Aurora and vent fields. In all cases, white bars represent a distance of 50 cm, inserts are 2× to 4× magnified. Left colum Polaris (**A**) Large numbers of amphipods associate with the fresh basalt. (**B**) Flat areas are covered with hydrothermal channels, partly filled with in gravel. (**C**) Numerous small vents with coloured, filamentous bacterial mats and mineral precipitates. (**D**) Pillow lava is inhabited by assemblages of anenomes and sponges. (**E**) Bare rock covered with mineral precipitates and biogenic strcutures, likely bryzoans. Right column Aurora: (**F**) The venting area shows substantial black smokers and is covered with black (iron sulfide) and brown (iron oxide) precipitates. Swarms of amphipods inhabit this area (small insert). (**G**) Vent fissures are inhabited by filter feeders, sponge and anemones (**H**) Some vents release clear, hot waters, and have yellow and orange mats and precipitates. (**I**) Sediment-covered pillow lava at the flanks of the field are inhabited by glass sponges and shrimps. (**J**) Aurora sediments often show fissures and cracks with orange-brown precipitates.

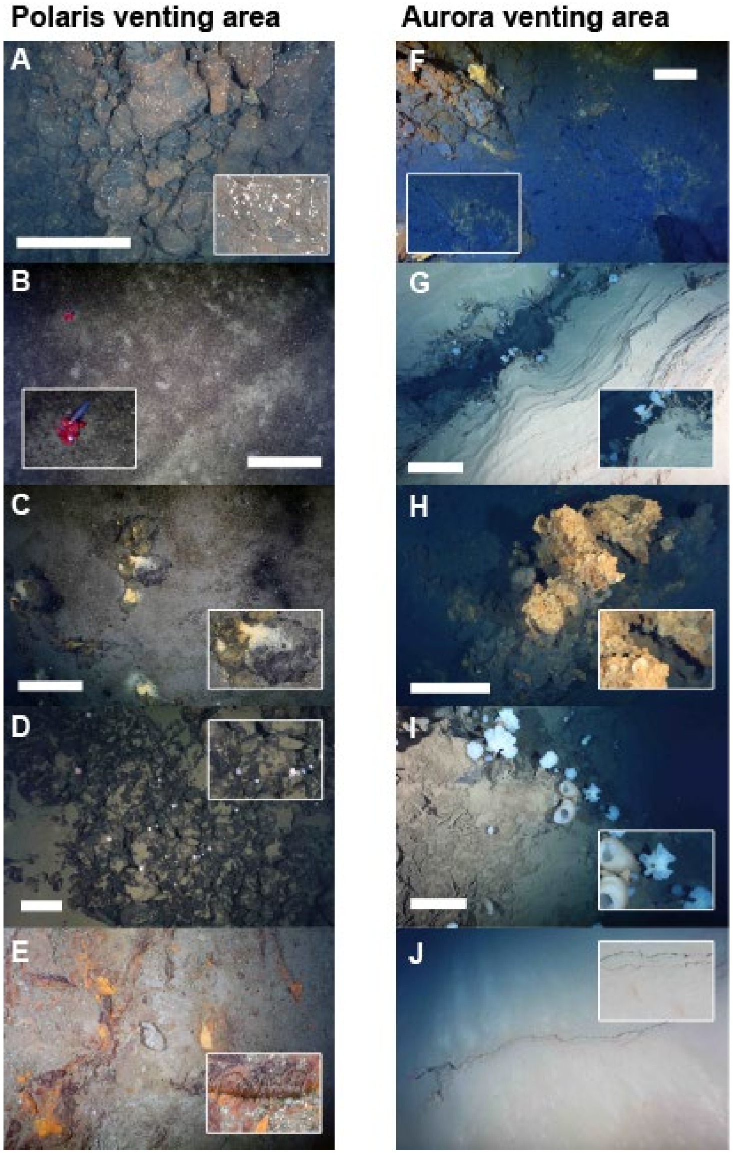

Supplementary Figure 2: Relationships between methane, hydrogen and ^{3}He in the Polaris and Aurora plume (**A**)**,** CH_4 vs. δ^3He with a highly linear relationship / conservative behavior of CH_4 in the water column. (**B**) H_2 vs. δ^3He with less hydrogen than δ^3He anomalies showing consecutive hydrogen consumption. (**C,D,E**) H_2-CH_4 ratios vs. δ^3He values and methane, respectively. All three figures show decreasing hydrogen / methane ratios with decreasing plume signal = dilution of the plume. This shows a predominant consumption of hydrogen in the plume. Red circles represent samples with highest temperature anomalies i.e. from least diluted, buoyant plume samples. PS101. (**F**) Methane concentrations in the Aurora plume are mostly linear.

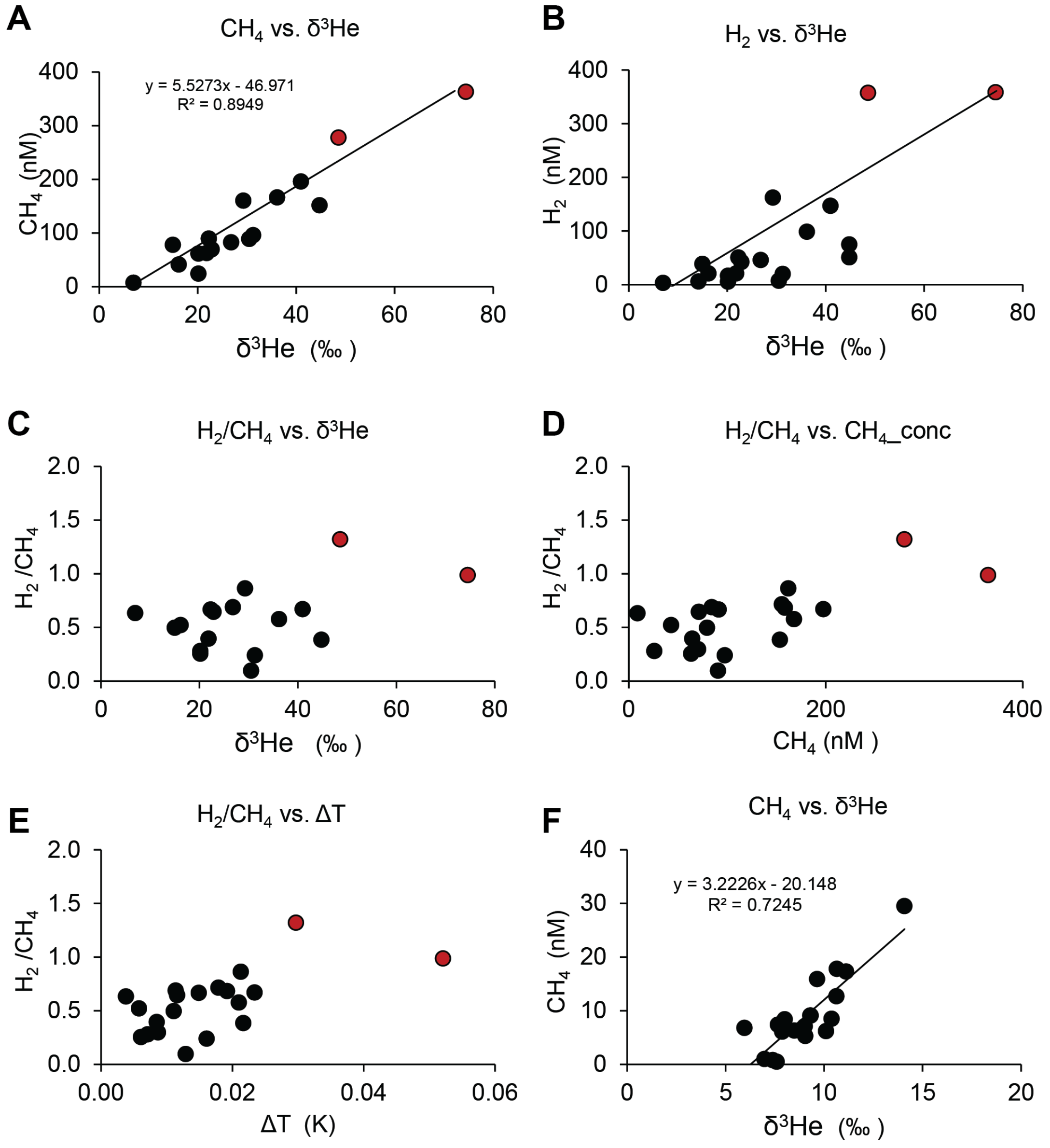

Supplementary Figure 3. Additional Hydrogen/ methane consumption experiments in the Polaris plume (see Fig. 5). All replicate bottles (9 to 21 per experiment) were prepared headspace-free and hydrogen/methane were added dissolved in sterile seawater. For each data point, an artificial air headspace was added to *n*=3 biological replicate bottles, and hydrogen and methane were measured in technical replicate from each bottle, Three data points had only 2 replicates, in 2 additional cases measurements could not be replicated. For single values see SourceData_Fig5_suppFig3 file**.** (**A**) PS101/188: In situ conditions, no additional methane/ hydrogen provided. (**B**) PS101/177: Both, hydrogen and methane were added with 150 nM final concentration at day 1. (**C**) PS101/159: Initially in situ conditions, after 2.5 days the remaining bottles received 200 nM H_2. (**D**) PS101/23. All replicates received 800 nM of hydrogen. (**E**) PS101/139; no gas addition; no consumption of hydrogen and methane. (**F**) Addition of hydrogen to a plume and to an above-plume water sample. First measurement after 30 h.

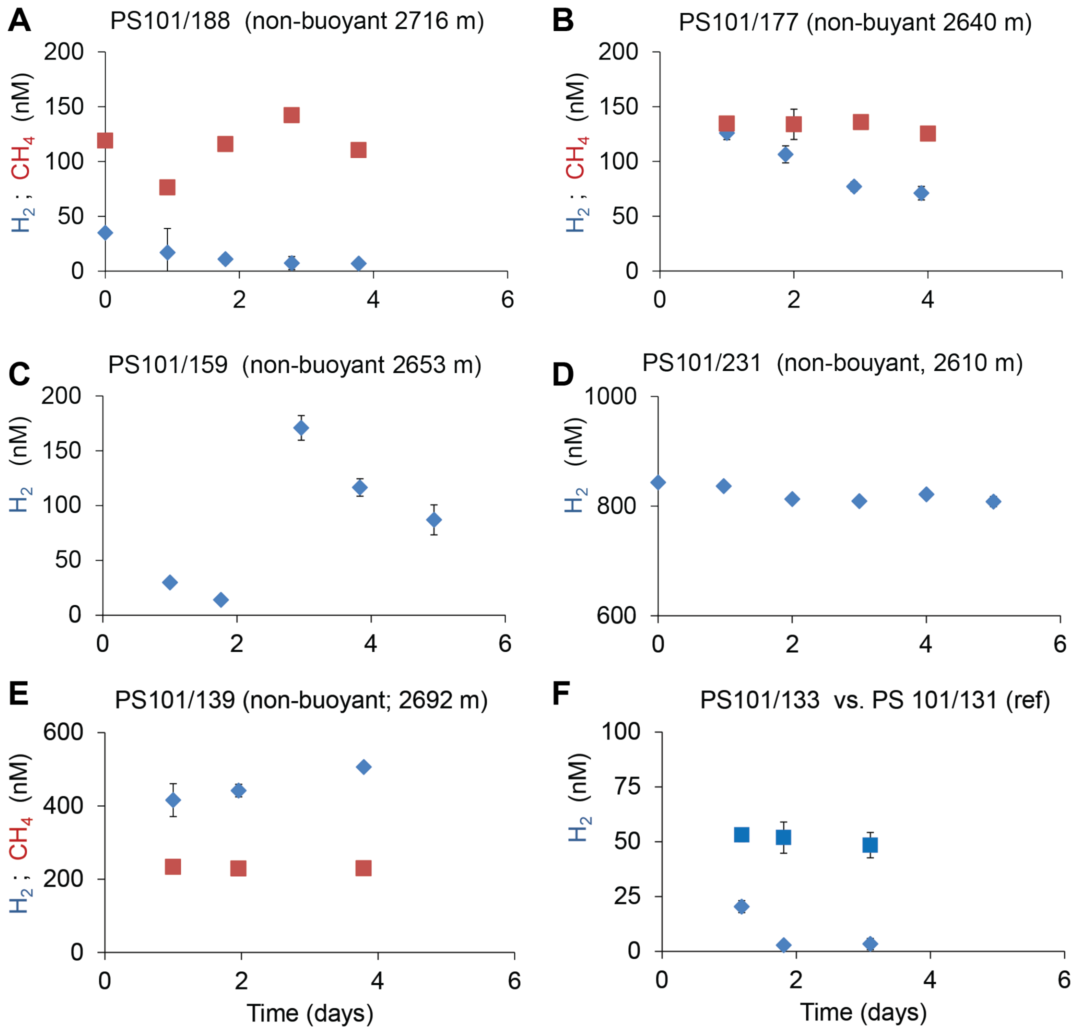

Supplementary Figure 4: Relative abundance 16S rRNA transcripts in the different water masses of Polaris and Aurora. In brackets: numbers of analyzed samples and sum of all listed taxa.

	Polaris				Aurora	
	Reference (7; 50%)	Above plume (2; 54%)	Plume (6; 72%)	Bottom water (4; 53%)	Above plume (3; 71%)	Plume (3; 90%)
Sulfurimonas *	0.23	2.77	13.32	0.73	39.85	73.39
SUP05 cluster	1.35	12.71	27.69	0.77	4.50	8.28
SAR202 clade, uncultured bacterium	9.82	6.78	4.30	3.47	7.45	2.19
SAR202 clade, uncultured Chloroflexi bacterium	5.45	3.78	2.24	1.77	4.22	1.22
Marinimicrobia, SAR406 clade, uncultured bacterium	5.82	5.36	2.73	3.65	3.03	0.76
SAR202 clade, uncultured deep-sea bacterium	2.83	2.01	1.16	1.06	2.03	0.63
Methylomonaceae, Milano-WF1B-03	0.16	0.73	1.42	0.05	0.99	0.53
SAR202 clade, hydrothermal vent metagenome	1.71	1.13	0.80	0.71	1.63	0.44
SAR202 clade, uncultured sediment bacterium	1.80	1.07	0.72	0.86	1.08	0.39
SAR202 clade, uncultured Chloroflexi bacterium HF4000_28F02	1.79	1.13	0.75	0.99	1.10	0.37
PAUC34f, uncultured bacterium	2.11	1.81	0.78	0.85	1.26	0.31
Pseudomonas	0.66	0.27	0.99	0.28	0.09	0.26
Microtrichaceae, Sva0996 marine group	1.39	1.07	0.64	0.77	0.76	0.24
Nitrosopumilaceae, uncultured marine crenarchaeote SAT1000-49-D2	1.27	0.86	0.41	0.57	0.19	0.17
Marinimicrobia, SAR406 clade, Marinimicrobia bacterium SCGC AAA003-E22	1.46	1.63	0.71	0.72	0.69	0.15
Methylomonaceae, Marine Methylotrophic Group 2	3.55	0.76	0.60	0.35	0.15	0.15
Arctic97B-4, marine group, uncultured bacterium	1.06	0.76	0.42	0.93	0.58	0.15
Ciliophora, Intramacronucleata, Conthreep *	2.24	0.85	0.96	11.19	0.27	0.11
Ciliophora, Intramacronucleata, Spirotrichea	0.65	0.39	0.56	2.42	0.26	0.08
Ciliophora, Intramacronucleata, Litostomatea	0.36	0.18	0.21	1.32	0.20	0.05
SAR11 clade, Clade I, Clade Ia	0.12	0.13	0.06	0.23	0.03	0.02
Colwellia *	1.38	0.82	1.26	8.76	0.04	0.02
Solimonas	0.13	0.00	0.07	0.00	0.00	0.01
Solimonadaceae, uncultured	0.14	0.00	0.10	0.00	0.00	0.01
Acidovorax	0.28	0.00	0.01	0.00	0.04	0.01
Lentimonas	1.08	0.37	0.35	1.22	0.02	0.01
Zhongshania *	0.04	0.51	0.54	0.07	0.00	0.00
Labyrinthulomycetes, uncultured eukaryote *	0.21	0.12	0.26	2.65	0.02	0.00
Psychrobium *	0.38	0.08	0.14	1.45	0.01	0.00
Foraminifera, Globothalamea	0.06	0.03	1.53	0.02	0.01	0.00
Brevundimonas *	0.16	0.03	0.11	0.02	0.01	0.00
Labyrinthulomycetes, uncultured marine eukaryote	0.01	0.00	0.01	0.01	0.00	0.00
Diatomea, Coscinodiscophytina	0.09	0.01	0.03	0.03	0.00	0.00
Profundimonas *	0.09	0.07	0.20	1.40	0.00	0.00
Labyrinthulomycetes, uncultured labyrinthulid	0.16	0.09	0.23	2.56	0.00	0.00
Methyloversatilis *	0.01	1.45	0.31	0.05	0.00	0.00
Vannellida, Platyamoeba *	0.04	0.32	0.69	0.19	0.00	0.00
Methylotenera *	0.00	0.44	0.36	0.08	0.00	0.00
Methylophaga *	0.02	0.49	2.14	0.06	0.00	0.00
Rhodococcus *	0.02	0.06	0.48	0.13	0.00	0.00
Aurantimonas *	0.00	0.10	0.61	0.03	0.00	0.00
Thalassolituus *	0.03	2.35	0.70	0.35	0.00	0.00

Supplementary Figure S4. Comparison of similarity of microbial communities within and between in-plume and out-plume samples. (**A**) nMDS based on Bray-Curtis similarity calculated on 16S rRNA gene (V3−V4 region) counts (Hellinger transformed). (**B**) nMDS based on Jaccard similarity calculated on 16S rRNA gene (V3−V4 region) counts (PA transformed). (**C**) nMDS based on Bray-Curtis similarity calculated on 16S and 18S rRNA reads (cDNA) counts (Hellinger transformed). (**D**) nMDS based on Bray-Curtis similarity calculated on reconstructed genes transcripts counts (Hellinger transformed). Analysis of similarity (ANOSIM) is reported in Supplementary Table 4.

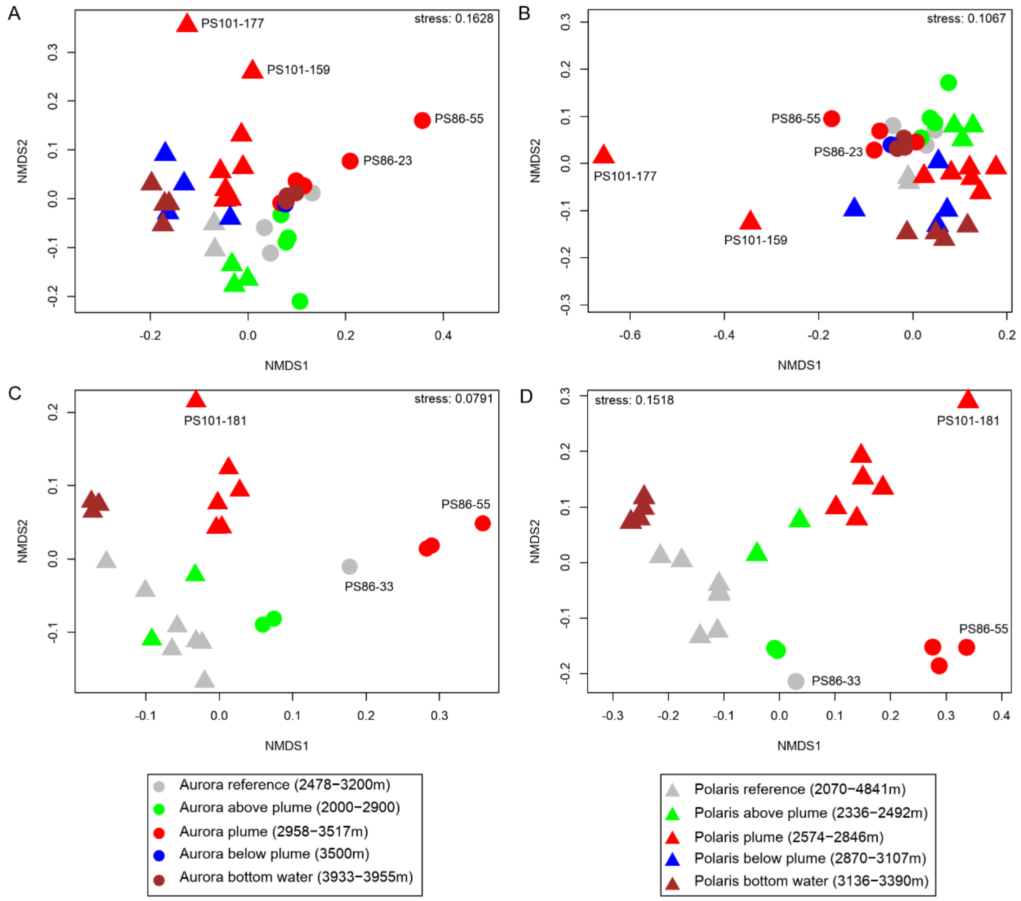

Supplementary Figure 5. Statistical analysis of bacterial diversity of different water types collected at Aurora and Polaris mounds. (**A**), Species richness as number of OTUs vs water type, (**B**) exponential Shannon index (**C**) inverse Simpson index of samples from the different water types. REF: reference; AP: above plume; P: plume; BP: below plume; BW: bottom water.

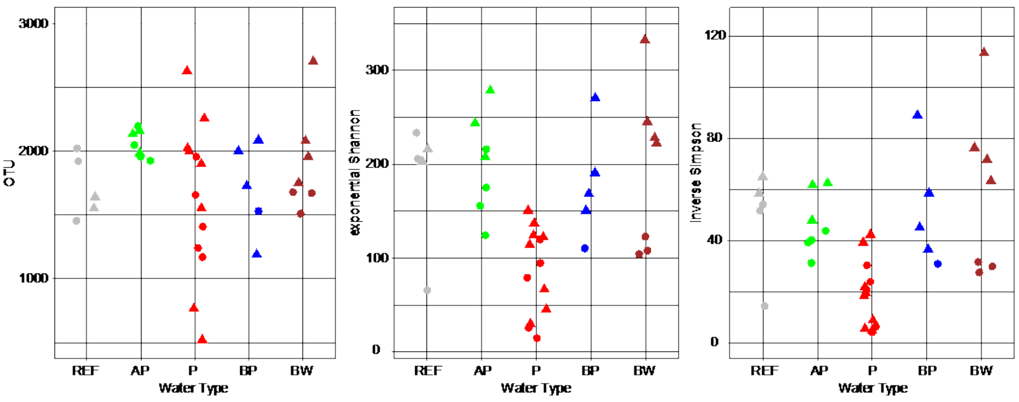

Supplementary Figure 6: Phylogenetic analysis of SUP05 bacteria from the two vent sites and other locations based on the 16SrRNA gene.

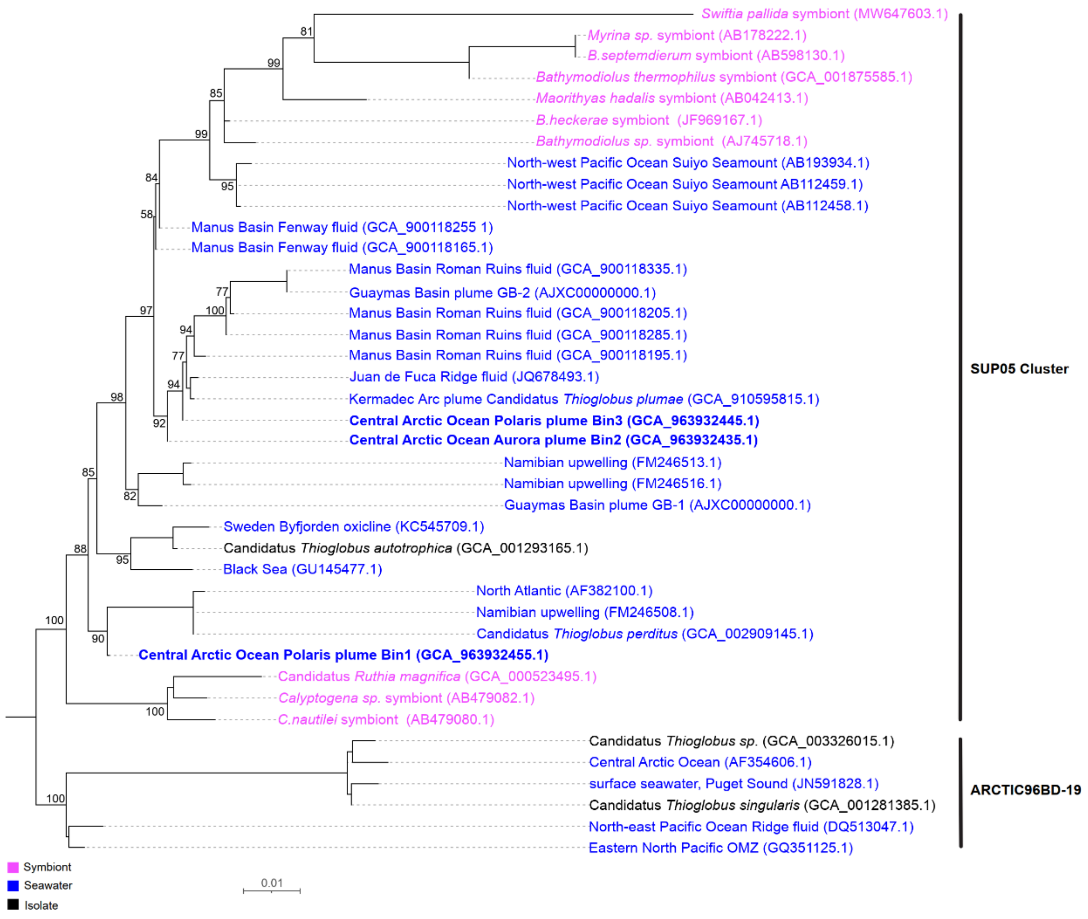

Supplementary Figure 7. Phylogenetic analysis of the NiFe hydrogenase of Ca. S. pluma and SUP05 bacteria.

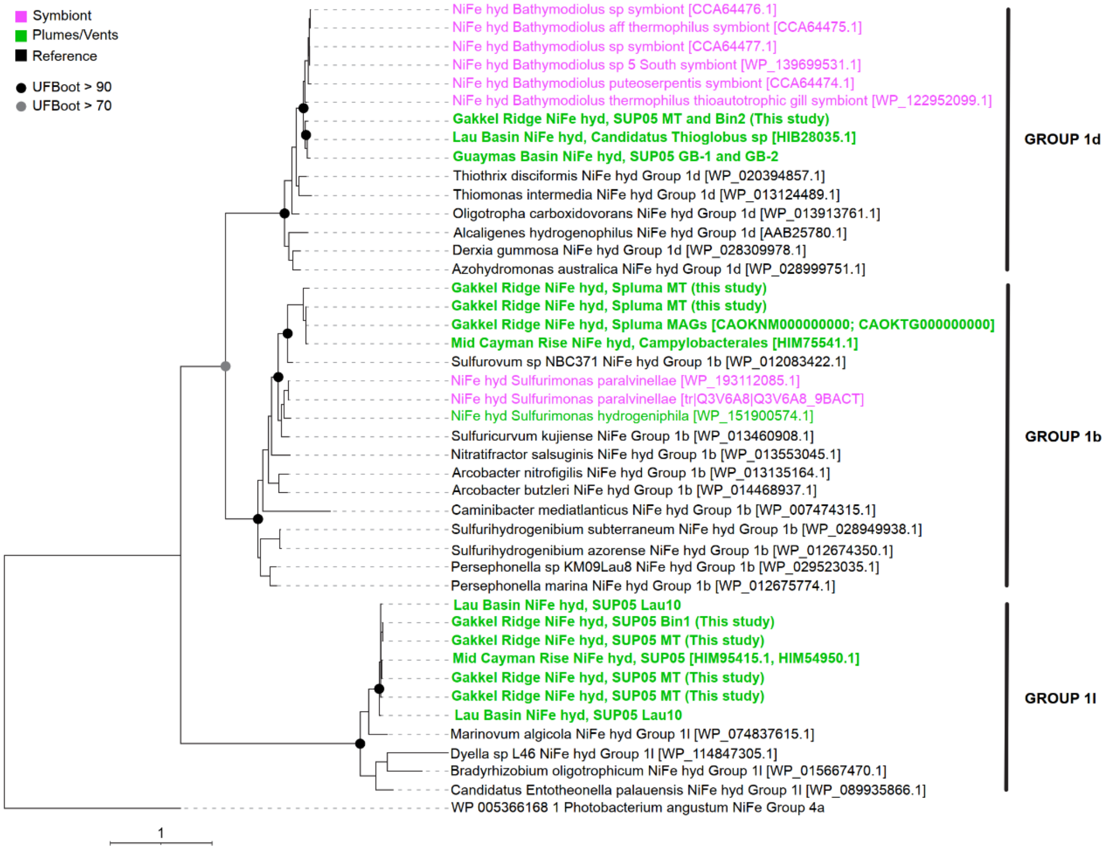

Supplement: Supplementary file 1 [file Data_Sheet_1.PDF]
